# Catalyst-Free Synthesis of a Mechanically Tailorable, Nitric-Oxide-Releasing Organohydrogel and Its Derived Underwater Superoleophobic Coatings

**DOI:** 10.1021/acsami.4c21695

**Published:** 2025-03-20

**Authors:** Aasma Sapkota, Arpita Shome, Natalie Crutchfield, Joseph Christakiran Moses, Isabel Martinez, Hitesh Handa, Elizabeth J. Brisbois

**Affiliations:** † School of Chemical, Materials, & Biomedical Engineering, University of Georgia, Athens 30602, Georgia, United States; ‡ Pharmaceutical and Biomedical Sciences Department, College of Pharmacy, University of Georgia, Athens, Georgia 30602, United States

**Keywords:** organohydrogel, nitric oxide, cytocompatibility, antibacterial, underwater superoleophobic coating

## Abstract

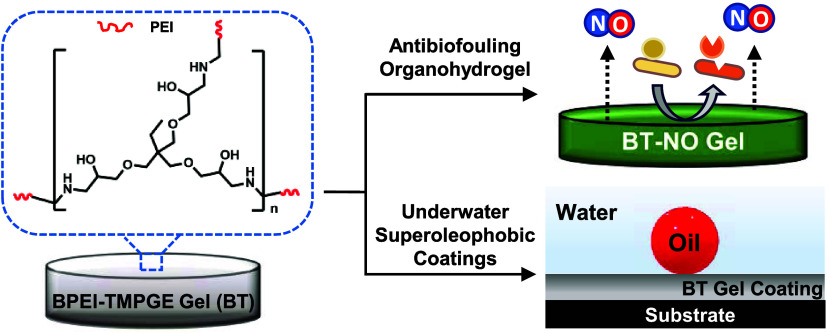

Organohydrogels are an emerging class of soft materials that mimick the mechanical durability and organic solvent affinity of organogels and the biocompatibility and water swelling ability characteristics of hydrogels for prospective biomedical applications. This work introduces a facile, catalyst-free one-step chemical approach to develop an organohydrogel with impeccable antibiofouling properties following the epoxy-amine ring-opening reaction under ambient conditions. The mechanical properties of the as-fabricated organohydrogel can be tailored depending on the concentration of the epoxy-based cross-linker, from 0.10 to 1.12 MPa (compressive modulus). The affinity of the as-developed organohydrogel to both organic solvents and water was exploited to incorporate the antimicrobial nitric oxide donor (NO) molecule, S-nitroso-*N*-acetylpenicillamine (SNAP) from ethanol, and subsequently, the water-sensitive NO-releasing behavior of the organohydrogels was analyzed. The SNAP-incorporated organohydrogels release physiologically active levels of NO with 3.13 ± 0.27 × 10^–10^ and 0.36 ± 0.14 × 10^–10^ mol cm^–2^ min^–1^ flux of NO release observed at 0 and 24 h, respectively. The as-reported organohydrogel demonstrated excellent antibacterial activity against *Escherichia coli* and *Staphylococcus aureus* with >99% and >87% reduction, respectively, without eliciting any cytotoxicity concerns. Moreover, the organohydrogel with remarkable water uptake capacity was extended as a coating on different medically relevant polymers to demonstrate transparent underwater superoleophobicity. Thus, the facile synthesis of the reported organohydrogel and its derived underwater antifouling coating can open avenues for utility in biomedical, energy, and environmental applications.

## 1 Introduction

Gels are physically or covalently cross-linked, three-dimensional semisolid systems with an entrapped liquid phase that display variable mechanical properties.^1^ Based on the type of liquid (organic solvent/aqueous phase) involved in the fabrication, gels can be categorized as hydrogels or organogels.^2^ Hydrogels are water-based gel systems with a resemblance to biological tissues, whereas organogels entrap organic solvents as their liquid phase. Hydrogels are desired for biomedical applications due to their high content of water, flexibility, softness, and biocompatibility.^3^ However, conventional single network hydrogels that excel in many biological aspects often show poor mechanical strength. Although tough hydrogels formed with double interpenetrating networks, nanocomposite fillers, microspheres, etc. have been reported, the practical utility of hydrogels is still limited due to the inability to use organic solvents, poor affinity toward hydrophobic compounds, and limited choice of gelators.^4,5^ Contrary to water-based hydrogels, organogels are mechanically robust, organic solvent-derived systems with applications in sensors, anti-icing, antifouling, droplet manipulation, food processing, and drug delivery.^6,7^ One of the major limitations that restricts the translation of organogels for extensive biological applications is the associated cytotoxicity stemming from the gelators or organic solvents. To overcome the challenges encountered by organogels and hydrogels and yet capitalize on the advantages of each of these gel systems, organohydrogels were introduced.

Organohydrogels are gel networks made up of hydrophilic, hydrophobic, or a combination of these polymer systems in a binary water–organic solvent system.^8^ Organohydrogels possess the benefit of both systems with biocompatibility and water swelling aspects of hydrogels and robust mechanical/storage durability and hydrophobic molecule affinity of organogels. This qualifies organohydrogels as excellent candidates for use in stretchable sensors,^9^ ionic skins,^10^ wound dressing,^11^ and anticounterfeiting.^12^ Particularly, the ability to use binary solvent systems has sparked increasing interest in developing antifreezing and conductive organohydrogels.^2^ A few reports have also explored the antimicrobial aspects of organohydrogels.^13,14^ Bao et al. reported an antifreezing and antibacterial conductive organohydrogel using one-dimensional (1D) silk nanofibers and two-dimensional (2D) graphitic carbon nitride in a poly(vinyl alcohol) system with uniformly dispersed aluminum chloride.^9^ The presence of Al^3+^ in this organohydrogel matrix exhibits >99% antibacterial characteristics against *Escherichia coli* and *Staphylococcus aureus*. Liang et al. reported a thermoplastic recycled gelatin-oxidized starch, glycerol, and zinc chloride comprising organohydrogel exhibiting thermoenhanced supercapacitor and sensor applicability along with antibacterial properties.^14^ Song et al. utilized chestnut tannin, silver nanoparticles, and aluminum chloride in a water–glycerol system to obtain a conductive organohydrogel with antifreezing and antibacterial characteristics.^15^

Most of the organohydrogel systems exploit the presence of metal ions to exhibit antibacterial characteristics; however, toxicity concerns owing to the leaching of the metal ions limit its practical applicability.^16−18^ Reports on loading antimicrobial drugs or gasotransmitters in organohydrogel systems to further explore the biological benefits remain unprecedented in the literature. Moreover, the reported antibacterial organohydrogels synthesis is a time-consuming multistep reaction with freeze/thaw or heat/cool cycles alongside temperatures varying from −20 to 90 °C.^9,15^ Additionally, the translation of three-dimensional (3D) organohydrogels to coatings on medical-grade polymers would realize the potential for real-world applicability as antifouling coatings on medical devices. Thus, there is a need to develop novel cytocompatible and antibacterial organohydrogels for biomedical applications, following facile fabrication processes without requiring harsh catalysts.

*N*-Diazeniumdiolates (NONOates) and *S*-nitrosothiols (RSNOs) such as *S*-nitroso-*N*-acetylpenicillamine (SNAP) and *S*-nitrosoglutathione (GSNO) are extensively recognized gasotransmitter donor molecules that release nitric oxide (NO) to exhibit impeccable antibacterial properties.^19−21^ As a small gaseous molecule, NO can easily permeate through microbial membranes and cause damage through the formation of reactive oxygen species (ROS).^22^ Additionally, as a gasotransmitter with a short half-life, NO can be the solution to the ever-growing antimicrobial resistance issue that is commonly observed with the use of conventional antibiotics.^23^ Nitric-oxide-releasing hydrogels have been widely reported;^24,25^ however, the lack of mechanical investigation and limited choice of NO-donor molecules owing to aqueous solubility concerns limits the applicability. Grayton et al. developed a pluronic F127 organogel for the release of cyclodextrin-based NO donor for increased *in vitro* skin permeation of NO and to achieve *in vivo* reduction in tumor growth,^25^ without any investigation on the durability of the organogel. Reports of durable NO release and cytocompatible organohydrogels are rare in the literature. Hence, there exists immense potential in combining the (a) bioactive NO donors with (b) durable, cytocompatible, and water–organic solvent compatible organohydrogel systems to develop novel 3D-polymeric gel networks for biomedical applications. Further, the translation of NO-releasing organohydrogel systems into antifouling coatings on a wide range of substrates is desirable for combating bacterial contamination in medical devices.

Herein, a catalyst-free chemical approach for synthesizing a mechanically durable organohydrogel for prospective biomedical applications as a bulk material and a substrate-independent coating is introduced. The ring-opening reaction between the amine functionalities of branched polyethylenimine (BPEI) and epoxy groups of trimethylolpropane triglycidyl ether (TMPGE) results in the formation of an organohydrogel network within 2 h without the need for any catalyst. The ability of the as-developed organohydrogel to take up both organic solvents and the aqueous phase proved useful for the incorporation of a nitric-oxide-releasing small molecule and further exploring its biological applicability after aqueous preconditioning. This is the first report of a NO-donor-loaded, cytocompatible organohydrogel that exhibited excellent antibacterial activity for both Gram-positive (*S. aureus*) and Gram-negative (*E. coli*) strains. Moreover, the durable organohydrogel can be extended as a substrate-independent coating on different polymeric substrates without compromising the optical transparency to exhibit underwater superoleophobicity. Thus, we anticipate that the current organohydrogel and its derived underwater antifouling coating can open avenues for utility in biomedical, energy, and environmental applications.

## 2 Experimental Section

### 2.1 Materials

Branched polyethylenimine (BPEI), trimethylolpropane triglycidyl ether (TMPGE), *N*-acetylpenicillamine (NAP), tetrahydrofuran (THF), sodium nitrite (NaNO_2_), ethylenediaminetetraacetic acid (EDTA), hydrochloric acid (HCL), sulfuric acid (H_2_SO_4_), oil red, MTT (3-[4,5-dimethylthiazol-2-yl]-2,5-diphenyl tetrazolium bromide) assay, and dichloromethane (DCM) were purchased from Sigma-Aldrich (St. Louis, MO 63103). Polycaprolactone (PCL) was purchased from Thermo Fisher Scientific (Waltham, MA). Sylgard 184 silicone elastomer base and curing agent were purchased from Dow Silicone Corporation (Midland, MI). Elasteon 5-325 was purchased from Biomerics (Salt Lake City, UT). Dulbecco’s modified Eagle’s medium (DMEM) and trypsin–EDTA were purchased from Corning (Manassas, VA 20109). Penicillin–streptomycin (Pen–Strep) and fetal bovine serum (FBS) were obtained from Gibco-Life Technologies (Grand Island, NY 14072). The bacterial strains *S. aureus* (ATCC 6538) and *E. coli* (ATCC 25922) and NIH/3T3 mouse fibroblast cells were purchased from the American Type Culture Collection (ATCC, CRL-1658). Phosphate-buffered saline (PBS) 0.01 M, pH 7.4, used for *in vitro* experiments, containing 138 mM NaCl, 2.7 mM KCl, and 10 mM sodium phosphate, was purchased from Sigma-Aldrich (St. Louis, MO 63103). Luria–Bertani (LB) broth and LIVE/DEAD BacLight bacterial viability kit were obtained from Fisher Bioreagents (Fair Lawn, NJ). LB Agar was purchased from Difco Laboratories Inc. (Detroit, MI). All of the buffers and media were sterilized in an autoclave at 121 °C, 100 kPa (15 psi) above atmospheric pressure for 30 min before biological studies.

### 2.2 Fabrication and Characterization of Organohydrogels

#### 2.2.1 Synthesis of *S*-Nitroso-*N*-Acetylpenicillamine

*S*-nitroso-*N*-acetylpenicillamine (SNAP) was synthesized by modifying a previously established protocol.^26^ Briefly, *N*-acetylpenicillamine (NAP) was dissolved in methanol in the presence of concentrated HCl and H_2_SO_4_. Then, aqueous NaNO_2_ was added dropwise to the mixture to nitrosate the solution. This solution was kept in the dark in an ice bath for 8 h to allow for the precipitation of SNAP crystals. Finally, SNAP crystals were collected by vacuum filtration, and unreacted nitrites were removed by vigorously washing the product with DI water. The product was then dried overnight in a desiccator to remove any trace solvent and stored at −20 °C in the dark until further use.

#### 2.2.2 Nuclear Magnetic Resonance

The purity of synthesized SNAP was confirmed through ^1^H NMR using a Bruker Ascend 400 MHz spectrometer. For NMR analysis, 7 mg of SNAP was dissolved in dimethyl sulfate-d6 (DMSO-d6), and the purity was determined through integration of the peaks obtained from the scan.

#### 2.2.3 Fabrication of Organohydrogels

The BPEI- and TMPGE-derived organohydrogels (abbreviated as BT gels) were synthesized through an epoxy-amine ring-opening reaction. First, BPEI and TMPGE were added to a glass vial in the required proportions. Then, ethanol was added to the vial, and the solution was vortexed for 30 s. Thereafter, the solution was allowed to solidify under ambient conditions for 2 h. For developing the NO-releasing BT gels, either 20 or 30 mg/mL of SNAP resulting in ∼5 or 7.5 wt % of SNAP was added to the BPEI-TMPGE mixture in ethanol and sonicated for 1 min. Afterward, the solution was allowed to solidify under ambient conditions. For all subsequent experiments, the as-fabricated organohydrogels were punched out into a 6 mm disk using a hole puncher. For biological studies, BT_1_ and BT_1_-NO_30_ organohydrogels were conditioned in PBS (pH 7.4) at 37 °C for 2 h before experiments to remove trace ethanol and unreacted amines.

### 2.3 Organohydrogels Characterization

#### 2.3.1 Attenuated Total Reflectance-Fourier Transform Infrared (ATR-FTIR) Spectroscopy

Attenuated total reflectance-Fourier transform infrared (ATR-FTIR) spectra of synthesized organohydrogels and their precursors were recorded using a Spectrum Two spectrometer from PerkinElmer (Greenville, SC) with an ATR accessory equipped with a Ge diamond crystal. The spectra of the organohydrogel mixture at 0 and 2 h were scanned from 4000 to 650 cm^–1^ with 32 scans and a resolution of 4 cm^–1^.

#### 2.3.2 Water Swelling Ratio

The water uptake ability of the organohydrogels was tested by submerging the samples in DI water for 24 h at room temperature and 37 °C. The dry weight of the sample was measured before incubation in water. At the end of 24 h, the organohydrogels were extracted from the water and excess unbound water was wicked off and weighed again. The percentage water swelling ability of the organohydrogels was calculated using the following formula, where *W* represents the weight
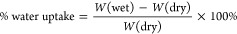


#### 2.3.3 Scanning Electron Microscopy (SEM) and Energy-Dispersive X-ray Spectroscopy (EDS)

The surface morphology of the BT organohydrogel was characterized by using scanning electron microscopy (SEM, FEI Teneo, FEI Co.). First, 10 nm of a gold–palladium coating was applied on the surface of the organohydrogel samples using a Leica EM ACE200 sputter coater (Buffalo Grove, IL). Then, the samples were scanned for surface characteristics by subjecting them to an accelerating voltage of 10.00 kV. Energy-dispersive X-ray spectroscopy (EDS, Oxford Instruments), an extension of SEM, was used to detect and quantify the presence of various elements on the surface of the gel.

#### 2.3.4 Mechanical Testing

To systematically investigate the effects of varying concentrations of functional amines present, mechanical testing was conducted. A modified version of ASTM D695 was followed for the evaluation of the mechanical properties of organohydrogel samples. Uniaxial compression testing was performed by a Mark-10 series 5 force gauge (Mark-10 Copiaque, NY). The organohydrogels were compressed to failure at a rate of 1.0 mm/min. The mechanical testing was completed with cylindrical samples (approximately 6 mm in diameter and 6 mm in height). Each specimen’s diameter and thickness were measured to the nearest 0.025 mm at several points. The minimum value of the cross-sectional area and length was recorded for each specimen. A total of 6 samples were compressed for each formulation of the organohydrogels. The force curves from uniaxial compression were used to determine the modulus of compression, compressive strength, and compressive toughness. The modulus of the organohydrogel was calculated by finding the slope of the linear (elastic) region of the stress–strain curves, and the compressive strength is the maximum strain applied to the organohydrogel before fracture. The regions of strain used to find the compressive modulus were between 0.2 and 0.3 and the linearity was confirmed by using *R*^2^ values ≥ 0.99. The compressive toughness was calculated from the area under the curve of the stress–strain plots up to the point of failure.

### 2.4 NO-Release Kinetics

The NO release from SNAP-loaded BT_1_ and BT_5_ organohydrogels was measured with a Sievers chemiluminescence nitric oxide analyzer (NOA 280i, Zysense, Boulder, CO). Prior to the NO release study, the organohydrogel samples were incubated in PBS (pH 7.4) for 2 h for conditioning. The NO release from the organohydrogels was characterized in the presence of PBS with 100 μM EDTA (pH 7.4), as EDTA can chelate any catalytic NO release from the presence of metals. The organohydrogels were wrapped in a Kim wipe moistened with PBS before recording the NO release in dark conditions at 37 °C to mimic physiological-like conditions. For real-time NO analysis, the NOA cell was partially submerged in a water bath at 37 °C. The NOA cell with PBS + EDTA was then continuously purged with nitrogen gas to facilitate the movement of any produced NO into the reaction chamber, where NO reacts with ozone to produce nitrogen dioxide in an excited state (NO^2*^). The excited nitrogen dioxide then decays to produce a photon, which is detected by PMT to calculate the amount of NO being released from the sample. The measured NO release is normalized to the total surface area of the gel.

### 2.5 Storage Stability

The stability of the organohydrogel was assessed by storing BT_1,_ BT_1_-NO_30_, BT_5_, and BT_5_-NO_30_ samples at 4 and 37 °C in dry conditions. At the end of 24 h and 7 days, the mechanical property of the samples was evaluated through compression testing. The compressive strength and modulus of the organohydrogel samples were determined in the same manner as Section 2.3.4.

Additionally, to determine the functionality of the SNAP-containing samples post storage, the NO release from BT_1_-NO_30_, BT_5_, and BT_5_-NO_30_ was measured. The NO release study was carried out in moist conditions after 2 h of conditioning, as mentioned in Section 2.4.

### 2.6 *In Vitro* Antibacterial Activity

#### 2.6.1 Planktonic Bacteria Quantification

To determine the antimicrobial efficacy, a 0.6 cm diameter organohydrogel was punched into a disk with a surface area of *ca.* 1 cm^2^. Then, the organohydrogels were conditioned in 1 mL of PBS at 37 °C for 2 h. The antibacterial activity of the organohydrogels was determined against *E. coli* and *S. aureus* using a 24 h planktonic bacterial assay and zone of inhibition. Single isolated colonies of *E. coli* and *S. aureus* were inoculated in LB broth and incubated at 37 °C for 8 h at 150 rpm. The bacterial culture was centrifuged at 3500 rpm for 7 min to isolate the bacterial pellet that was then resuspended in PBS solution (pH 7.4). The bacterial solution was then diluted to obtain an optical density (OD) of 0.1 using a ultraviolet–visible (UV–vis) spectrophotometer (Cary 60, Agilent Technologies) at 600 nm wavelength equaling to a final bacterial concentration of ∼10^6^ colony forming units (CFU)/mL. To 1 mL of this diluted bacterial solution, UV-sterilized and conditioned organohydrogel samples were introduced. Then, the organohydrogels were incubated at 37 °C in a shaking incubator at 150 rpm for 24 h (*n* = 4), after which the solution was diluted and plated onto LB agar plates using a spiral plater (Eddy Jet 2, IUL Instruments). The agar plates were incubated at 37 °C for 24 h to allow for bacterial growth on the plates. At the end of 24 h, the bacterial colonies on the agar plates were counted using an automated colony counter (Sphere Flash, IUL Instruments). The CFUs of bacteria pertaining to the planktonic bacterial solution in which the organohydrogel was incubated were quantified. The percentage of reduction in bacterial viability was determined by the following equation (with respect to BT_1_ control), where *C* represents the concentration of viable bacteria in CFU
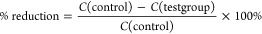


#### 2.6.2 Zone of Inhibition

For further analysis of the antibacterial ability of the organohydrogel, a zone of inhibition study was performed against *S. aureus* and *E. coli*. Individual colonies of bacteria were isolated and grown in LB broth for ∼8 h in a 37 °C incubator at 150 rpm. The bacteria solution was centrifuged to obtain a pellet, as mentioned in Section 2.6.1. Afterward, the pellet was resuspended in LB broth to obtain ∼10^6^ CFU/mL of bacterial solution. This solution was spread on an LB plate, and a sterilized filter paper (control), BT_1_ and BT_1_-NO_30_ samples were placed on the plates and gently pressed against the agar using a sterile tweezer. The plates were allowed to dry for 10 min in the biosafety cabinet under ambient conditions before placing them in the incubator at 37 °C for overnight incubation. Afterward, the zone of inhibition was determined by measuring the diameter of the zones where bacterial growth was absent. The result from the study is reported as diameter ± standard deviation (SD, *n* = 4).

#### 2.6.3 Live/Dead Assay

A single isolated colony of *S. aureus* was inoculated in LB broth and incubated at 37 °C for 8 h while being shaken at 150 rpm. The bacteria colony was then centrifuged and diluted to obtain an OD of 0.1 at 600 nm as described in Section 2.5. Then, BT_1_ and BT_1_-NO_30_ samples were incubated in the bacteria solution for 24 h at 37 °C and 150 rpm. The live/dead assay dye was prepared by adding 10 μL of SYTO9 and 20 μL of propidium iodide (PI) to 10 mL of DI water to yield a working dye concentration of 6 μM SYTO9 (which stains all bacteria green) and 30 μM PI (stains dead bacteria red). Then, the organohydrogels incubated in the bacterial solution were incubated in the dye solution for 15 min in dark conditions. Afterward, the organohydrogel samples with adhered stained bacteria were observed using a fluorescence microscope (Advanced Microscopy Group’s EVOS FL Fluorescence Imaging Microscope (AMG, Mill Creek, WA)) and imaged.

### 2.7 *In Vitro* Cytotoxicity Assay

#### 2.7.1 MTT Assay

The cytocompatibility of BT_1_ and BT_1_-NO_30_ organohydrogels was assessed by an indirect contact cytotoxicity MTT assay following the ISO 10993 standard. A cell culture treated 24-well plate was used to seed 3T3 mouse fibroblast cells at a seeding density of 5 × 10^4^ cells/mL culture medium (25,000 cells/cm^2^) and the plate was incubated at 37 °C for 24 h with 5% CO_2_ to obtain subconfluency of 80% before testing the effect of leachate. The next day, the samples (BT_1_ and BT_1_-NO_30_; *n* = 5) were UV-sterilized for 30 min in a biosafety cabinet and then conditioned in DMEM for 2 h. Following the ISO standards (Biological evaluation of medical devices; Part 5: Tests for in vitro toxicity), the organohydrogels were added to a 24-well plate containing an insert to maintain a barrier between the organohydrogel and the cells. The aspect ratio for the extraction of the leachate from the tested organohydrogel was followed based on ASTM F619–20 (Standard Practice for Extraction of Materials Used in Medical Devices), where 1 mL of media was added per 1 cm^2^ of sample submerged through the cell culture insert. The samples were allowed to interact with the cells for 24 h at 37 °C with 5% CO_2_. At the end of the incubation, the inset with samples was removed, and the media was pipetted out. Immediately, 100 μL of MTT solution (5 mg/mL) and 900 μL of DMEM were added to each well, and the plate was incubated at 37 °C for 3 h. Then, the solution was pipetted out, and the same volume of DMSO was added to each well followed by gentle rocking of the plate to dissolve any formazan crystals present in the well. A distinct color change was observed in the wells due to the formation of formazan crystals reduced from MTT by viable cells, which was quantified using a plate reader (Cytation 5 imaging multimode reader, BioTek). The absorbance (*A*) of the wells at 570 nm is reported in terms of the relative cell viability compared with the control cells
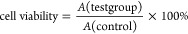


#### 2.7.2 Scratch Assay

To understand the effect of leachates from the organohydrogels on mammalian cells’ proliferative ability, an *in vitro* scratch assay was performed to assess whether the organohydrogels can be used for prospective wound healing applications. First, 3T3 mouse fibroblast cells were seeded into a cell culture insert (Ibidi, Fitchburg, Wisconsin) within a 24-well plate at 7000 cells per compartment of the inset. The insert consisted of two compartments with a divider that prevented the migration of cells from one compartment to another, maintaining a linear zone where cells were absent. The well consisting of the inset was then incubated at 37 °C with 5% CO_2_ for 24 h. At the end of 24 h, the divider was removed and the medium was replaced by diluted leachate (1:10 leachate:DMEM) from the organohydrogel samples (BT_1_ and BT_1_-NO_30_). The individual wells containing cells were monitored by using a phase-contrast EVOS-XL microscope at different time points. Additionally, pictures were taken at these time points to track cell migration, and ImageJ (Wayne Rasband, National Institute of Health) was used to quantify the reduction in the scratch area. A representative image was chosen for the figure panel, and the experiment was performed in triplicates.

### 2.8 Fabrication of Organohydrogel Coatings

BT coating (BT_1_) was fabricated on the surface of a wide range of substrates to exhibit superoleophobic properties under water. Polydimethylsiloxane (PDMS) substrate was prepared by mixing Sylgard 184 silicone elastomer base with curing agent at a 10:1 ratio and curing for 2 h at 100 °C. Similarly, PCL substrates were prepared by dissolving PCL in THF at a concentration of 50 mg/mL and casting the solution in a Teflon mold followed by overnight drying in ambient conditions. Additionally, Elasteon 5-325 (Elasteon from hereon) substrates were prepared by dissolving Elasteon pellets in THF at a concentration of 70 mg/mL and casting the solution in a Teflon mold followed by overnight drying in ambient conditions.

Thereafter, the BPEI and TMPGE mixture at the ratio of 5:4 was mixed in ethanol and then coated onto PDMS, PCL, and Elasteon surfaces through drop-casting. Around 50 μL of the organohydrogel precursor was applied on a ∼2 × 1 cm substrate and allowed to air-dry for 2 h to obtain the coating.

### 2.9 Underwater Superoleophobicity

The underwater contact angle of the gel-coated substrates was investigated using an Ossila Contact Angle Goniometer (Sheffield, U.K.). Coated samples were taped to glass slides and placed under water for the assessment of superoleophobicity. A 20 μL droplet of DCM stained with Oil Red was pipetted onto the coated surface, and digital images as well as contact angle images were taken. Using droplet edge detection and polynomial fitting, the contact angle was determined for each sample using the Ossila Contact Angle (v3.0.3.0) software.

### 2.10 Statistical Analysis

All experiments in this study are conducted with a sample size of *n* ≥ 4. Data are reported as the mean ± standard deviation (SD). All statistical analyses were performed using Prism 9.1 (GraphPad Software, San Diego, CA). Statistical comparisons of treatment groups against control groups were analyzed using two-way ANOVA with Tukey’s method for correcting multiple comparisons. Bacterial statistical analysis was performed on the logarithms of CFUs for each treatment. Values of *p* < 0.05 were deemed statistically significant.

## 3 Results and Discussion

### 3.1 Fabrication and Characterization of Organohydrogels

**Figure 1:**
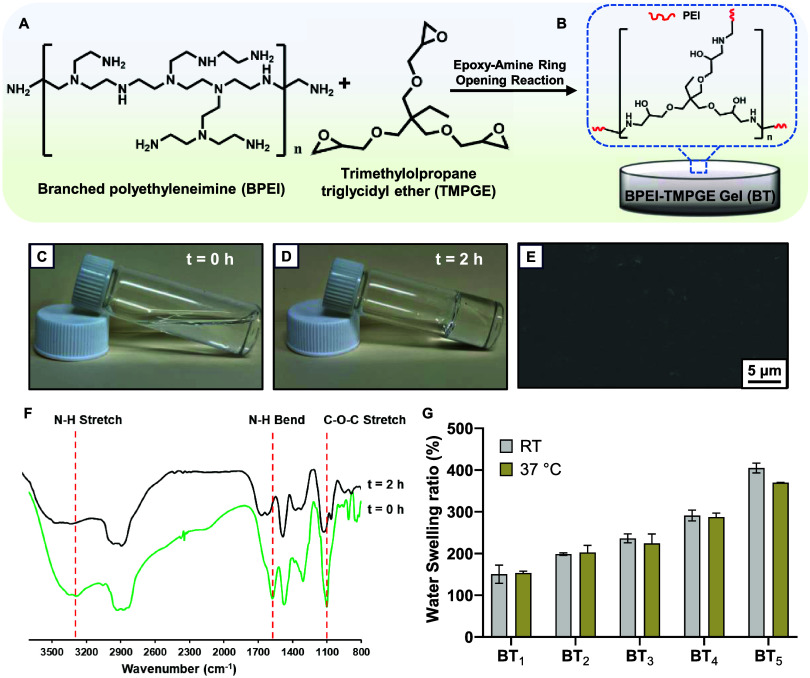
(A) Schematic depicting branched polyethylenimine (BPEI) and trimethylolpropane triglycidyl ether (TMPGE) undergoing an epoxy-amine ring-opening reaction. (B) Formation of BT organogel with cross-linked epoxy and amine groups. BT gel mixture at (C) hour 0 and (D) hour 2. (E) Surface of the BT_1_ gel observed through SEM, (F) FTIR spectra of the gel precursor and cross-linked gel (BT_1_) showing the difference in the peak intensity. (G) Water swelling ratio of various BT organohydrogels (BT_1_–BT_5_) at room temperature and at 37 °C.

Epoxy-amine reactions are popular for the fabrication of polymeric networks in industrial settings due to the tunability of the system.^27^ Deriving from their excellent thermal stability, tunable glass transition temperature, and degradability, epoxy-amine systems have been applied in a broad range of applications such as maintenance coating, automobile adhesives, and binders for composites.^28^ Capitalizing on the robust epoxy-amine reaction, in the current work, the amine groups present in BPEI act as nucleophiles that can open the epoxy rings of TMPGE through the SN_2_ reaction to form a three-dimensional organohydrogel as shown in Figure 1A–D.^29^ The as-developed organohydrogel can be used as a standalone organohydrogel for use in drug delivery, wound healing, or large-scale antifouling applications without requiring extreme temperatures, long reaction time, or any other external catalysts.^30^ Organohydrogels with a wide range of mechanical properties were developed by varying the concentration of BPEI as tabulated in Table S1 to obtain BT_1_, BT_2_, BT_3_, BT_4_, and BT_5_.

The progress of the epoxy-amine reaction to fabricate the organohydrogel was monitored through ATR-FTIR analysis. The reaction mixture of BPEI-TMPGE at *t* = 0 h exhibited (i) N–H stretch at 3288 cm^–1^, (ii) N–H bend at 1619 cm^–1^ corresponding to BPEI, and (iii) the C–O–C epoxy ring stretch at 1055 cm^–1^ as shown in Figure 1F (green spectra). After the organohydrogel formation at *t* = 2 h, a decrease in the intensity of the N–H stretch, N–H bend, and C–O–C stretch was observed, indicating the successful covalent reaction between the amine and epoxy groups as shown in Figure 1F (black spectra). Similar peaks and simultaneous reduction in peak intensities were observed for the other compositions of the organohydrogels, *i.e.*, BT_1_–BT_5_ (Figure S1A). Additionally, for the SNAP-incorporated sample, BT_1_-NO_30_, the FTIR spectra exhibited carbonyl stretching peaks at 1658 and 1741 cm^–1^ corresponding to the presence of amide and carboxylic groups in SNAP.^31^ The presence of the intense peak at 2970 cm^–1^ can be attributed to the sp^3^ C–H stretching for the methyl groups present in SNAP (Figure S1B).

The SEM image of the organohydrogel surface showed a smooth surface, insinuating the surface homogeneity (Figure 1E). The organohydrogel exhibited a nonporous solid structure with no indication of phase separation as expected. Elemental analysis showed the presence of C, N, and O on the surfaces of both BT_1_ and BT_5_ with higher nitrogen content on BT_5_, which can be attributed to the higher amine content (Figure S2A,B). Additionally, the water swelling ratio of the organohydrogels with increasing concentrations of BPEI was tested as the swelling capacity would indicate the ability of the organohydrogel to uptake nutrients and metabolites, drug diffusion rate, blood and exudate absorption, etc.^32^ The swelling ability was determined at room temperature and 37 °C to observe the effect of physiological temperature on the swelling of the gels. The water swelling ratio seemed to increase with the increase in the concentration of BPEI in BT organohydrogels (Figure 1G). The presence of amine groups introduces hydrophilicity in the organohydrogels, facilitating enhanced water uptake and resulting in a higher swelling ratio. Notably, there was no significant difference in the water swelling ratio for respective organohydrogels that were incubated at room temperature and 37 °C. Thus, the current organohydrogel mimics the water swelling ability of hydrogels.

### 3.2 Investigation of Mechanical Properties

In recent years, mechanically robust hydrogels that can withstand persistent load-bearing are gaining interests for use at medical settings.^33^ The combination of two key properties (i) strong mechanical properties for real-world application and (ii) the ability to mimic hydrogels for use in biological systems, makes organohydrogels desirable for translative research. Organohydrogel systems fabricated through the epoxy-amine reaction can produce mechanically variable organohydrogels with enhanced durability that closely mimics biological systems with hydrogel-like properties (Table S2). Through precise tailoring of its mechanical properties, these biocompatible organohydrogels can be applied to various tissue-matching modulus and soft robotics applications.^34^

**Figure 2:**
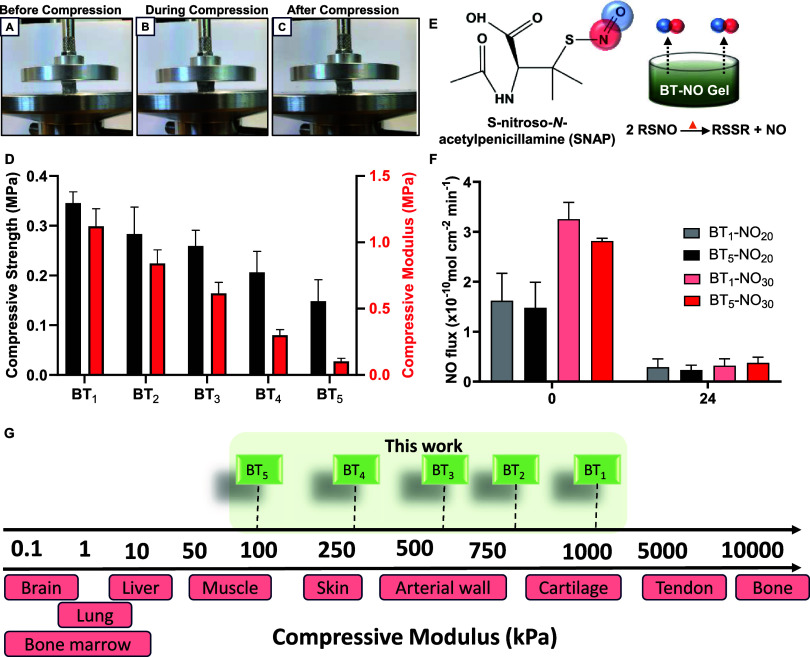
(A–C) Representative picture of the mechanical testing of the organohydrogel before, during, and after compression when the organohydrogel is subjected to 25% compression of its original height. (D) Compressive strength and compressive modulus of organohydrogel samples (BT_1_–BT_5_). (E) Chemical structure of NO-donor SNAP and its release mechanism from the gel. (F) NO release from organohydrogel samples BT_1_ and BT_5_ when different concentrations of SNAP (20 and 30 mg/mL) are loaded onto the gel. (G) Compressive modulus (kPa) of the proposed organogel when compared to various human tissues.

The mechanical properties of the organohydrogels with differing chemical compositions were investigated, wherein BT_1_–BT_5_ were compressed up to 25% strain and it was observed that the physical integrity of the organohydrogels remained undeterred, as demonstrated in Figure 2A–C. BT_1_ showed a higher modulus of elasticity and yield strength, while BT_5_ displayed a softer nature with the compressive modulus of 1.12 ± 0.13 MPa and 0.10 ± 0.02 MPa, respectively (Figures 2D and S3A). The wide range of mechanical properties of the organohydrogels corresponding to the compressive modulus closely mimics the mechanical properties of human tissue, making BT organohydrogel an ideal material for biological applications (Figure 2G).^35^ The BT_1_ organohydrogel with an amine to epoxy ratio of 5:4 exhibited a higher compressive modulus owing to the covalent cross-linking of most amines with epoxy. However, BT_5_ organohydrogel with an amine to epoxy ratio of 9:4 exhibited lower stiffness due to the presence of residual, uncross-linked BPEI amines. A similar trend was reflected in the compressive toughness as shown in Figure S3B, thus offering the feasibility to tailor the mechanics of the developed organohydrogel as desired. Additionally, when 30 mg/mL SNAP was incorporated into the organohydrogel, no significant difference was observed in the compressive modulus of BT_1_-NO_30_ and BT_5_-NO_30_ when compared to BT_1_ and BT_5_ (Figure S3C).

The organohydrogels fabricated in this study were tested for their ability to maintain mechanical integrity and functionality after storage in a refrigerator at 4 °C and 37 °C (physiological condition). The assessment of BT_1_ and BT_1_-NO_30_ gels stored at 37 °C showed a slight increase in both the compressive modulus and compressive strength at 24 h, which increased significantly on day 7, as shown in Figure S4A,B. On the other hand, BT_5_ showed an increase in the compressive modulus at 24 h after 37 °C storage without a significant change in the compressive modulus or strength for BT_5_ and BT_5_-NO_30_ after 7 days storage at 37 °C (Figure S4A,B). The increase in compressive modulus of BT_1_ and BT_1_-NO_30_ samples at 37 °C can be attributed to heat-induced catalysis of the epoxy-amine ring-opening reaction. Heat increases the kinetic energy of the molecules, facilitating higher rate of collision between amine and epoxy moieties, and resulting in a highly cross-linked material.^36^ This makes the gel stiffer and stronger.

For samples stored at 4 °C, a slight increase in compressive modulus was observed with BT_1_, BT_5_, and BT_1_NO_30_ at 24 h; however, there was no significant change in the compressive modulus at 7 days with any of the sample types (Figure S4C). Finally, for all of the samples stored at 4 °C, a significant increase in the compressive strength was observed when compared to fresh samples (Figure S4D). Minimal or no increase in compressive modulus and strength of BT_5_ and BT_5_-NO_30_ can stem from the presence of minimal epoxy groups (in comparison to amines), lowering the chances of heat-induced cross-linking. However, it is evident that there is no significant difference between BT_1_ and BT_5_ with and without SNAP, which further proves that the inclusion of SNAP does not affect the compressive strength and modulus of organohydrogel samples.

### 3.3 Analysis of NO-Release Kinetics

Organohydrogels fabricated using both organic solvent or water is a popular strategy to synthesize environmentally adaptable dual-solvent systems.^37^ Organohydrogels can encapsulate a higher concentration and a variety of drugs (both hydrophobic and hydrophilic) based on the solubility, molecular weight, and chemical structure.^2^ S-nitrosothiols (RSNOs) such as SNAP and GSNO have been explored in the past for their tailored release behavior from the polymeric matrix. The water-soluble GSNO is a bioavailable primary RSNO whereas the water-insoluble SNAP is a synthetic tertiary RSNO formulated from the amino acid penicillamine.^38^ Due to the enhanced stability of SNAP as a tertiary RSNO and high solubility in ethanol of approximately 30 mg/mL, SNAP was chosen as the NO donor to be incorporated into the organohydrogel. Additionally, as a NO donor, SNAP has shown excellent long-term NO release in various *in vitro* and *in vivo* experiments.^39^

The purity of the synthesized SNAP was determined through the integration of NMR peaks and was found to be 97.93% (Figure S5). The highest amount of SNAP that can be readily soluble in the organohydrogel precursor solution was found to be 30 mg/mL, with insolubility observed at higher concentrations (Figure S6A–C). SNAP releases NO in the presence of heat, light, or metal ions and under physiological conditions.^40^ The translation of SNAP-based NO-releasing systems from polymeric films to gel systems is limited, as SNAP is not readily dispersed or dissolved in an aqueous medium. Here, utilizing the binary solvent system, SNAP is easily incorporated into the organohydrogel matrix by dissolving it in an environmentally preferable solvent (fabricated by fermenting renewable sources), ethanol, which can extend the utilization of SNAP-based organohydrogels.^41^ To assess the NO release, 20 and 30 mg/mL of SNAP were incorporated into BT_1_ and BT_5_ organohydrogel resulting in BT_1_-NO_20_, BT_1_-NO_30_, BT_5_-NO_20_, and BT_5_-NO_30_ samples, respectively. The NO release from these organohydrogels was quantified in real time using a chemiluminescence NOA at 37 °C.

As expected, NO release from BT_1_-NO_30_ and BT_5_-NO_30_ samples was higher when compared to that from BT_1_-NO_20_ and BT_5_-NO_20_. At 0 h, the NO release from BT_1_-NO_20_ and BT_5_-NO_20_ was recorded at 1.62 ± 0.55× 10^–10^ and 1.48 ± 0.51× 10^–10^ mol cm^–2^ min^–1^, respectively. Similarly, the NO release from BT_1_-NO_30_ and BT_5_-NO_30_ was found to be 3.13 ± 0.27 × 10^–10^ and 2.82 ± 0.05 × 10^–10^ mol cm^–2^ min^–1^, respectively at 0 h (Figure 2F). The NO release patterns of BT_1_ and BT_5_ were similar, demonstrating no significant correlation between the epoxy-amine cross-linking and NO release in moist conditions. Although all four tested groups came near exhaustion at 24 h with depletion of loaded SNAP, the organohydrogels still released a physiologically relevant amount of NO ∼0.2 – 0.4 × 10^–10^ mol cm^–2^ min^–1^. This release pattern with an initial high release followed by subsequent lower NO levels for 24 h is similar to the drug release kinetics often seen with medical implants where early burst release is followed by slow continuous release of drugs.^42^ The BT_1_-NO organohydrogel with higher SNAP content, *i.e.*, BT_1_-NO_30_, was used for the antibacterial study as enhanced NO flux can demonstrate better antibacterial activity. Additionally, BT_1_ was selected for biological studies moving forward due to its enhanced mechanical properties. Prior literature has shown that hydrogels with similar levels of NO release have shown excellent antibacterial efficacy with no cytotoxicity concern.^42^

To evaluate the functionality of the samples after storage, organohydrogels stored at 4 °C and 37 °C for 7 days were tested for their NO release behavior. When NO release from BT_1_-NO_30_ and BT_5_-NO_30_ was measured in moist conditions after 24 h storage, no significant change in NO flux was observed when compared to fresh samples (Figure S7A). This shows that the organohydrogel samples are unaffected by 24 h storage at 4 °C or 37 °C. Additionally, the samples stored at 4 °C exhibited no change in NO release behavior even after 7 days of storage. On the other hand, a significant reduction in NO flux was observed with BT_1_-NO_30_ and BT_5_-NO_30_ samples stored at 37 °C for 7 days (Figure S7B). This is expected as RSNOs such as SNAP are prone to heat catalysis and can rapidly catalyze NO release at higher temperatures.^40^ Interestingly, BT_1_-NO_30_ showed higher retention of SNAP after 7-day storage at 37 °C when compared to BT_5_-NO_30_, which can be attributed to the higher cross-linking density and the increase in the modulus of elasticity.^43^

### 3.4 Investigating the Antibacterial Activity of Organohydrogels

The threat of bacterial infection is often amplified in hospitals and is especially dangerous for patients who already have some form of underlying illness. The US Center for Disease Control and Prevention has stated that almost 1.7 million hospitalized patients acquire healthcare-associated infections (HCAIs) annually.^44^ Additionally, with the increase in antimicrobial resistance (AMR), conventional antibiotics are proving futile, turning AMR into one of the principal public health problems of the 21st century.^45^ NO-releasing materials can be a solution to the impending issue of AMR as radicals from NO have been proven effective in antibiotic-resistant species of bacteria.^46^ Generally, metal nanoparticles used in the fabrication of antimicrobial organohydrogels raise cytotoxicity concerns. Hence, the current work on the durable epoxy-amine-derived, NO-releasing organohydrogel is an attractive cytocompatible and effective solution to the ever-growing threat of infection.

**Figure 3:**
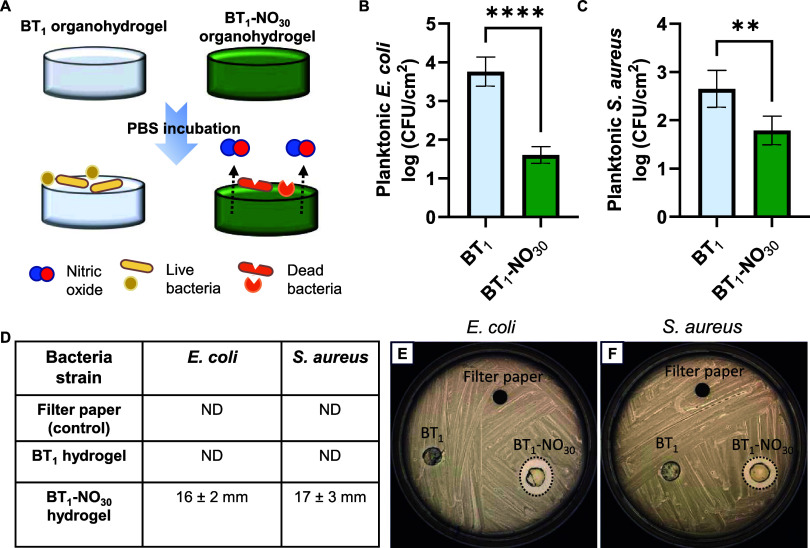
Antibacterial activity of organohydrogel samples against *E. coli* and *S. aureus*. (A) Schematic of antibacterial activity of the NO-releasing gel. Reduction in planktonic CFU of (B) *E. coli* and (C) *S. aureus* with the action of the NO-releasing gel. (D) Size of zone formation around control, BT_1_, and BT_1_-NO_30_ when tested against *E. coli* and *S. aureus*. Photograph of a Petri dish showing the formation of the zone of inhibition around BT-NO organohydrogel against (E) *E. coli* and (F) *S. aureus* (*n* = 4); * represents *p* ≤ 0.05, ** represents *p* ≤ 0.01, *** represents *p* ≤ 0.001, and **** represents *p* ≤ 0.0001. All data are represented as mean ± SD.

For the assessment of the antibacterial activity of the fabricated organohydrogel, the planktonic bacteria exposed to the organohydrogel were quantified through CFUs and normalized to the surface area of the gel. Inhibition of planktonic bacteria surrounding a medical implant is crucial as these bacteria eventually adhere on the surface of medical devices to form biofilms and worsen infection. As shown in Table S2, extensive biological studies with antibacterial organohydrogel have been lacking. For this study, both the control (BT_1_) and NO-releasing organohydrogel (BT_1_-NO_30_) were incubated in PBS for 2 h for conditioning (Figure 3A). Then, the antimicrobial activity of the organohydrogel was evaluated against *S. aureus* and *E. coli* through a 24 h bacterial assay with incubation at 37 °C. The results showed that the BT_1_-NO_30_ group had a significantly lower number of bacteria present in planktonic conditions when compared to BT_1_ in the cases of both *E. coli* and *S. aureus* (Figure 3B,C). BT_1_-NO_30_ showed a 99.42% and 87.31% reduction in *E. coli* and *S. aureus*, respectively, when compared to BT_1_. This result is in agreement with previous studies where NO-releasing polymeric substrates showed similar reduction in the viability of bacterial strains.^47^

Additionally, the antibacterial activity of the organohydrogels was tested through the formation of a zone of inhibition following 2 h of conditioning to demonstrate the ability of the organohydrogel to exhibit antibacterial activity in both aqueous and moist conditions. When tested along the filter paper (negative control) and the BT_1_ group, a distinct zone formation was observed around BT_1_-NO_30_ for *E. coli* and *S. aureus* (Figure 3E,F). The diameter of these zones was found to be 16 ± 2 mm and 17 ± 3 mm, respectively, for *E. coli* and *S. aureus*, whereas no zones were observed around the filter paper and BT_1_ organohydrogel (Figure 3D). To further investigate the antibacterial activity of the NO-releasing organohydrogel against the adhered *S. aureus*, live/dead stained *S. aureus* was imaged using a fluorescence microscope using SYTO9 that stains bacteria green, while PI specifically stains the dead bacteria red by binding to exposed DNA. Bacterial damage through membrane disruption results in the exposure of free DNA and cell debris from bacteria that tends to clump up together (Figure S8A,B).^48^ The membrane integrity of bacteria exposed to BT_1_-NO_30_ was compromised, resulting in more dead bacteria and clumped-up bacterial colonies (Figure S8C). It has been established that NO possesses broad-spectrum antibacterial properties due to its innate reactivity and formation of reactive byproducts that cause oxidative and nitrosative stress.^49^ Nitrosative species such as dinitrogen trioxide (N_2_O_3_) cause DNA deamination of bacterial cells, and the reaction of NO with superoxide, which is derived from bacterial respiration, results in the formation of peroxynitrite (ONOO^–^), eliciting DNA damage and lipid peroxidation.^50^ These mechanisms enable NO to significantly reduce the viability of bacteria without triggering any adverse toxicity.

### 3.5 Cytocompatibility Analysis

In recent years, cytocompatible organohydrogel-based systems have emerged to alleviate the preconceived notion of toxicity stemming from organogels.^51,52^ In particular, organohydrogels are favorable for the enhancement of the cytocompatibility of drug delivery systems. Here, the antibacterial activity of BT_1_-NO_30_ organohydrogel is promising, but it is important to establish the cytocompatibility of the components used in the fabrication of BT organohydrogel as well as the NO counterparts with healthy mammalian cells. The cytocompatibility of BT_1_ and BT_1_-NO_30_ organohydrogels was assessed by an indirect contact cytotoxicity MTT assay as well as through the collected leachates via scratch assay following the ISO 10993 standard.

**Figure 4:**
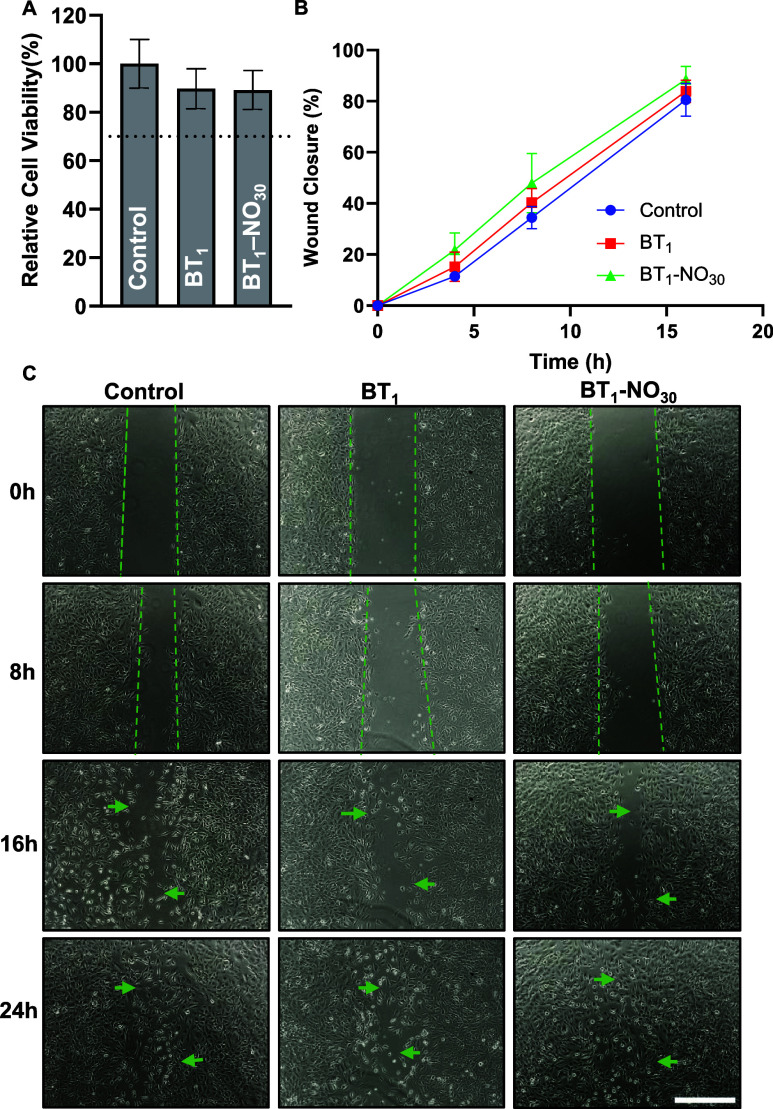
Cytocompatibility of organohydrogels evaluated against 3T3 mouse fibroblast cells. (A) Relative cell viability obtained through an indirect cell contact using the MTT cell viability assay. (B) Reduction of the scratch area subjected to different treatments, quantified using ImageJ. (C) Scratch area observed in wells treated with the leachate from BT_1_ and BT_1_-NO_30_ organohydrogel as well as blank DMEM (control). Scale bar represents 500 μm. All data are represented as mean ± SD (*n* ≥ 4).

The MTT assay examined the relative cell viability through indirect exposure of 3T3 mouse fibroblast cells through the enzymatic reduction of yellow 3-(4,5-dimethylthiazol-2-yl)-2,5-diphenyl-2H-tetrazolium bromide (MTT) to purple formazan in metabolically active cells that can be quantified via color intensity at 570 nm.^53^ All of the tested organohydrogel samples were found to have relative cell viability of >89%, which is above the required cytocompatibility threshold of 70% (Figure 4A).^54^ Previous studies of epoxy-amine networks have shown no cytotoxic effect of the network on other lines of mammalian cells.^55^ The results also support the previous cytocompatibility trend observed with the RSNO-incorporated gel matrix.^42,56^

To substantiate the cytocompatibility of the fabricated organohydrogel, the leachates obtained from each sample type were tested for cell migration and proliferation through the scratch assay. The concentration of SNAP in the diluted leachate solution of BT_1_-NO_30_ that was exposed to cells was found to be ∼0.30 mM/cm^2^ through UV–vis analysis (Figure S9). The leachates were collected over 24 h at 37 °C, exposed to cells growing on a 24-well plate, and monitored for 24 h (Figure S10). The images of cells in individual wells with different leachate treatments were taken using a microscope at various time points to demonstrate the role of leachates in cell migration *in vitro*.^57^ The assay showed that the leachates from BT_1_ and BT_1_-NO_30_ samples did not interfere with cell proliferation and migration when compared to the untreated control (cells exposed to normal culture media). It is pertinent to note here the NO flux noted for BT_1_-NO_30_ was 2.82 ± 0.05 × 10^–10^ mol cm^–2^ min^–1^, which is ∼2.5 fold more than the physiological levels seen in the endothelium (1.0 ± 0.05 × 10^–10^ mol cm^–2^ min^–1^); yet, these NO flux levels did not significantly affect the cell viability.^58^ As a gasotransmitter, NO has pleiotropic roles in maintaining many cellular functions, among which maintaining immunohomeostasis is very crucial. We envisage the organogel coatings shown here would initially help in wading off any implant-associated infection by the rapid NO release (Figure 2F) and the resultant NO flux (as noted in BT_1_-NO_30_ at 24 h to be ∼0.5 × 10^–10^ mol cm^–2^ min^–1^) will be conducive to maintain a healthy immune environment, preventing any adverse foreign body reaction by modulating the local immune response. This NO flux is in accordance with an earlier report where ∼3500 × 10^–12^ mol cm^–2^ s^–1^ (∼0.5 × 10^–10^ mol cm^–2^ min^–1^) flux levels significantly reduced fibrotic encapsulation in subcutaneously implanted biomaterials.^59^

Additionally, when cell migration observed at different time points was quantified using ImageJ, no significant difference was seen between the control and test groups (Figure 4B). By the 24 h time point, all test groups (control, BT_1_, and BT_1_-NO_30_) showed complete closure of the scratch area through the formation of uniform monolayer cells (Figure 4C). All test groups showed similar trends of cell migration and propagation at different time points, which emphasizes that the solvent system used or the polymer branches present in the organohydrogel network did not hinder the cell activity (Figure S11). This result is in agreement with previously reported studies that showed that possible leaching of NO donors from hydrogel beads does not have a detrimental impact on cell migration.^42^ This is advantageous as the organohydrogel system developed here can be used for prospective drug delivery or wound healing systems by incorporating drugs or bioactive molecules that could enhance the bioactivity of the organohydrogel system. Dual release systems from the NO-releasing organohydrogels (which showed antibacterial properties as discussed in Section 3.4) could be developed, which can curtail implant-associated infections, while the incorporation of a secondary cargo to modulate tissue function in regenerative medicine application opens the feasibility to further explore this organohydrogel.

### 3.6 Organohydrogel-Derived Underwater Superoleophobic Coatings

**Figure 5:**
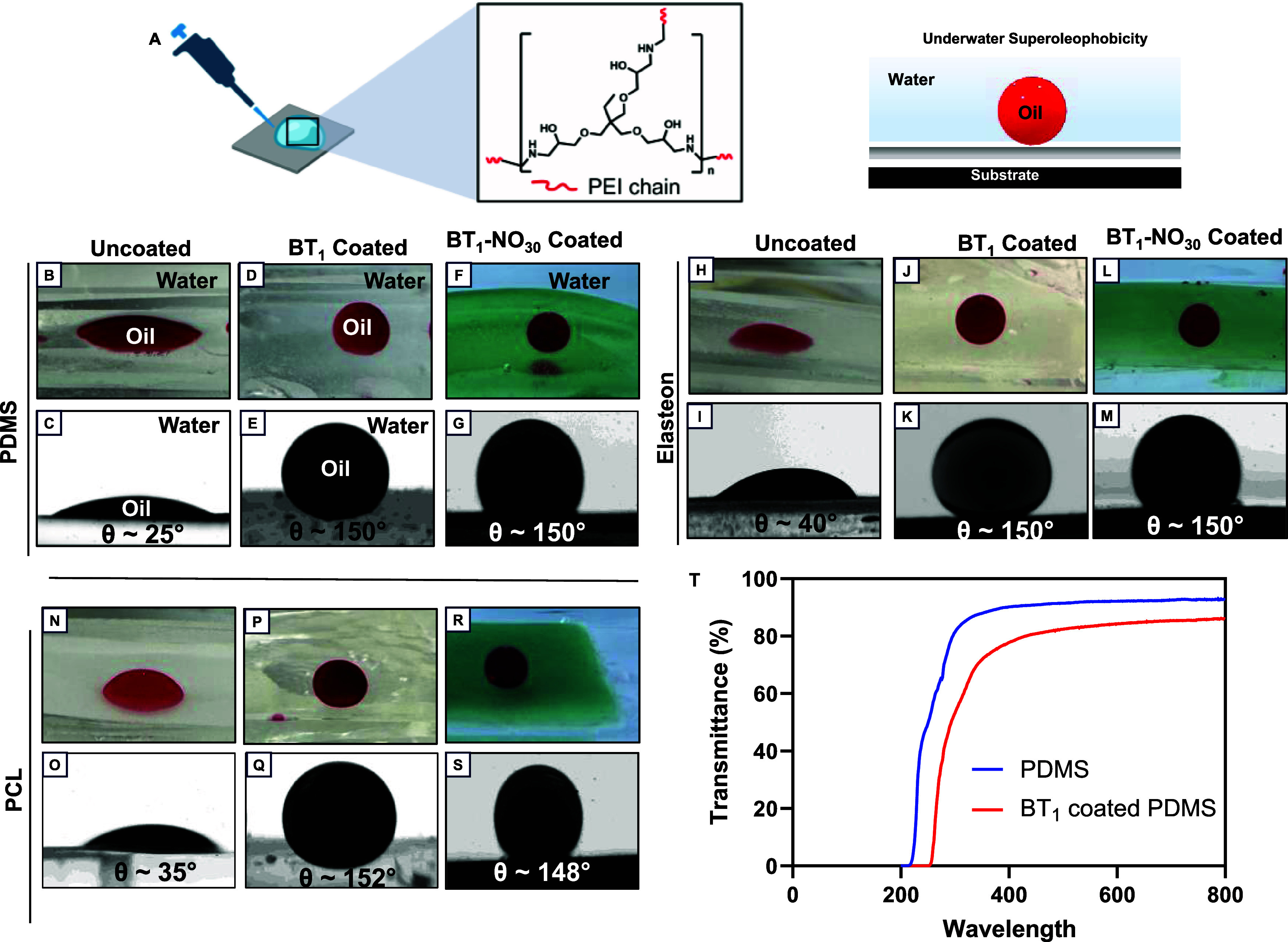
(A) Schematic of the BT_1_ organohydrogel being drop-casted on a substrate to prepare an underwater superoleophobic surface. Digital images of (B) uncoated, (D) BT_1_-coated, and (F) BT_1_-NO_30_-coated PDMS underwater with a DCM droplet. Contact angle images of (C) uncoated, (E) BT_1_-coated, and (G) BT_1_-NO_30_-coated PDMS underwater with a DCM droplet. Digital images of (H) uncoated, (J) BT_1_-coated, and (L) BT_1_-NO_30_-coated Elasteon underwater with a DCM droplet. Contact angle images of (I) uncoated, (K) BT_1_-coated, and (M) BT_1_-NO_30_-coated Elasteon underwater with a DCM droplet. Digital images of (N) uncoated, (P) BT_1_-coated, and (R) BT_1_-NO_30_-coated PCL underwater with a DCM droplet. Contact angle images of (O) uncoated, (Q) BT_1_-coated, and (S) BT_1_-NO_30_-coated PCL underwater with a DCM droplet. (T) Transmittance of BT_1_-coated PDMS compared to PDMS at a visible light wavelength.

Underwater superoleophobicity is a fish-scale-inspired liquid antiwetting state wherein the fish scales made up of calcium phosphate, proteins, and a thin layer of mucus gives it an overall high surface energy, *i.e.*, hydrophilicity.^60^ It is because of the presence of this hydrophilicity that a stable aqueous layer can be trapped within the fish scales to help it survive in oil/oily contaminated water.^61^ Over the years, underwater superoleophobicity has gained widespread prominence as antibacterial and antiplatelet adhesion surfaces owing to the presence of the stable aqueous layer that inhibits the adhesion of biofoulants.^62^ Generally, metal oxides, polyelectrolytes, and hydrogels (with high water swelling rate) have been used for constructing these passively antifouling underwater superoleophobic surfaces.^63^ However, the conventional stability issues associated with hydrogels remain a major concern for practical applications. Thus, in this work, the highly water-swellable hydrogel-like but mechanically stable organohydrogel was extended as an underwater superoleophobic coating on different prospective medically relevant polymers. With their excellent optical, electrical, and mechanical properties along with exceptional biocompatibility, polymers such as PDMS, PCL, Elasteon, etc., have been the elastomers of choice for the fabrication of medical devices such as catheters, bandages, dressings, implants, microvalves, optical systems, etc.^64,65^ However, these polymer-based devices suffer from serious fouling problems stemming from microbial and protein adsorption.^66^ Following a drop-casting method (Figure 5A), the as-developed organohydrogel with its excellent water uptake capacity imparts underwater superoleophobicity to the coatings on medical polymers such as PDMS, PCL, and Elasteon owing to the presence of the stable hydration layer that can aid in inhibiting the adhesion of biomolecules or microbes on the surface.^67^ As shown in Figure 5B,C, H,I, and N,O, the commercially procured medical polymers exhibited underwater oleophilicity with an OCA less than 40°. However, upon coating with the epoxy-amine-derived organohydrogel, different polymeric substrates exhibited underwater superoleophobicity with an OCA greater than 150° as shown in Figure 5D,E, J,K, and P,Q. Similarly, for the SNAP-incorporated organohydrogel, BT_1_-NO_30_ was extended as coating on different polymeric substrates with underwater OCA of ∼150° (Figure 5F,G, L,M, and R,S). This shows that the incorporation of SNAP into the coating does not affect the antifouling property of the coating and can exert additional antibacterial effect through NO release. The average thickness of the coating was found to be ∼ 47 μm when observed under SEM (Figure S12). The optical transparency of the organohydrogel-coated PDMS surface was found to be ∼86% (normalized with respect to uncoated PDMS with transmittance ∼ 92%), as shown in Figure 5T. The optical transparency of the coating opens additional avenues for utilizing the current organohydrogel coating in applications involving optical stability and uncompromised visibility.^68,69^ Thus, the ease of application and substrate-independent characteristics of the as-reported organohydrogel coating qualifies it for a wide range of applications ranging from antibiofouling, oil–water separation, biosensors, and wound dressing.^70^

## 4 Conclusions

The current work proposes the fabrication of a catalyst-free, three-dimensional organohydrogel that can also be translated as an underwater superoleophobic coating on a wide range of substrates. The cross-linking between the amine groups of branched polyethylenimine and the epoxy functionalities of trimethylolpropane triglycidyl ether under ambient conditions results in a mechanically durable organohydrogel with tailorable mechanical properties depending on the concentration of the epoxy-based cross-linker. The ability of the organohydrogel to swell both organic and aqueous phases allows the incorporation of hydrophobic small molecule (SNAP) and aqueous preconditioning for successful biological applications. The cytocompatible NO-releasing organohydrogel showed excellent antibacterial activity against *E. coli* and *S. aureus* with 99.42% and 87.31% reduction, respectively, in the viability of planktonic bacteria when compared to the organohydrogel without any SNAP incorporation. Furthermore, the organohydrogel was extended as an underwater superoleophobic coating on a wide range of polymeric substrates, including PDMS, Elasteon, and PCL with underwater–oil contact angles greater than 150°. Thus, the current chemical approach to develop a mechanically tailorable, biologically compatible organohydrogel and its derived underwater superoleophobic coatings can be expanded for biomedical, energy, and environmental applications.

## Supporting Information Available

Composition of various gel types and their corresponding compressive modulus and strength, FTIR of gels BT_1_–BT_5_, FTIR spectra of BT_1_-NO_30_; map sum spectra of BT_1_ and BT_5_ gels analyzed with EDS; compressive modulus of BT_1_, BT_1_-NO_30_, BT_5_, and BT_5_-NO_30_ organohydrogels, compressive toughness of BT_1_–BT_5_ organohydrogels, transmittance of BT_1_-coated glass substrate, representative stress–strain curve of BT_1_–BT_5_ organohydrogels; compressive modulus and compressive strength of samples stored at 4 and 37 °C for 7 days; ^1^H NMR spectra of *S*-nitroso-*N*-acetylpenicillamine (SNAP); BT_1_ gels with different SNAP concentrations; NO release from BT_1_-NO_30_ and BT_5_-NO_30_ gels after storage at 4 and 37 °C for 24 h and 7 days; schematic of the antibacterial mechanism of the BT_1_-NO_30_ organohydrogel, LIVE/DEAD of BT_1_ and BT_1_-NO_30_ gel against *S. aureus*; SNAP leaching from BT_1_-NO_30_ over 24 h and the corresponding calibration curve; schematic of the scratch assay experiment; cell images from the scratch assay over 24 h; and SEM cross section of the BT_1_-coated substrate (PDF)
