# Supplementary material for: Catalyst-Free Synthesis of a Mechanically Tailorable, Nitric-Oxide-Releasing Organohydrogel and Its Derived Underwater Superoleophobic Coatings
Source: ACS Appl Mater Interfaces. 2025 Mar 20;17(13):19335–47. doi: 10.1021/acsami.4c21695 (PMC11969437; doi:10.1021/acsami.4c21695)
Supplement: Supplementary file 1 — am4c21695_si_001.pdf [file am4c21695_si_001.pdf]

Supporting Information (SI)

Catalyst-Free Synthesis of a Mechanically Tailorable, Nitric Oxide Releasing Organohydrogel and its Derived Underwater Superoleophobic Coatings

Aasma Sapkota[a], Arpita Shome[a], Natalie Crutchfield[a], Joseph Christakiran Moses[a], Isabel Martinez[a], Hitesh Handa[ab], Elizabeth J. Brisbois[a]*

[a]School of Chemical, Materials, and Biomedical Engineering, University of Georgia, Athens 30602, United States

[b]Pharmaceutical and Biomedical Sciences Department, College of Pharmacy, University of Georgia, Athens, GA 30602, United States

**** For correspondence, E-mail: ejbrisbois@uga.edu***

Table S1. Composition of various gel types and their corresponding compressive modulus and strength

Gel type	Composition	Compressive modulus (MPa)	Compressive strength (MPa)
BT_1	**5:4**	1.12	0.35
BT_2	**3:2**	0.84	0.2
BT_3	**7:4**	0.62	0.26
BT_4	**2:1**	0.30	0.21
BT_5	**9:4**	0.10	0.15

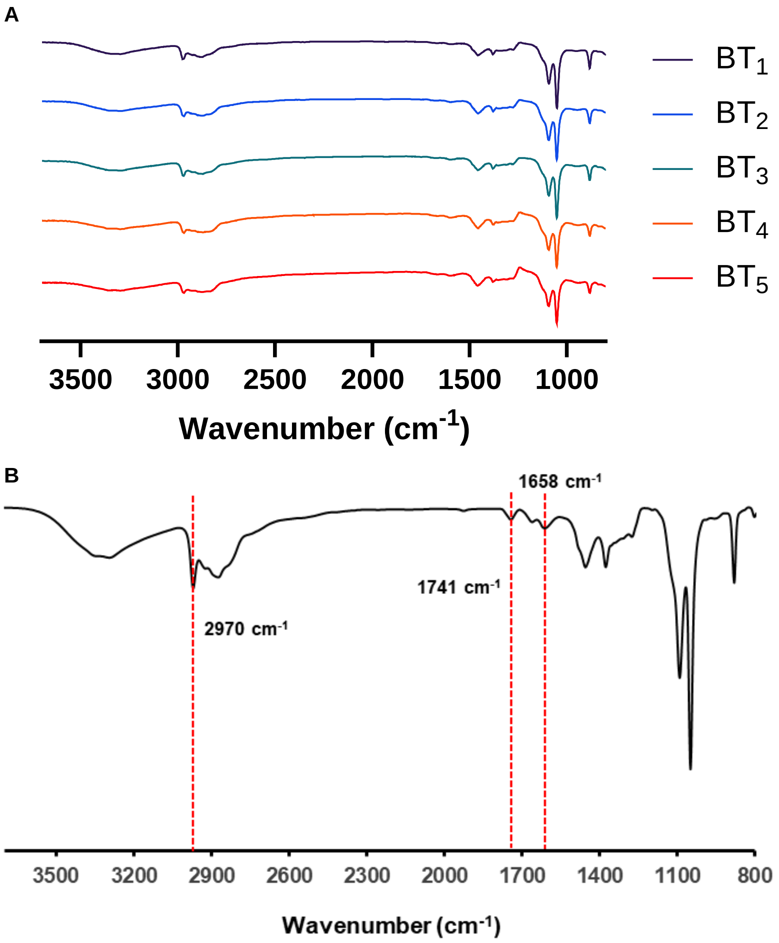

Figure S1. (A) FTIR spectra of BT gel samples (BT_1-BT_5) showing similar peaks. (B) FTIR spectra of BT_1-NO_{30} (BT_1 gel with 30 mg/mL SNAP).

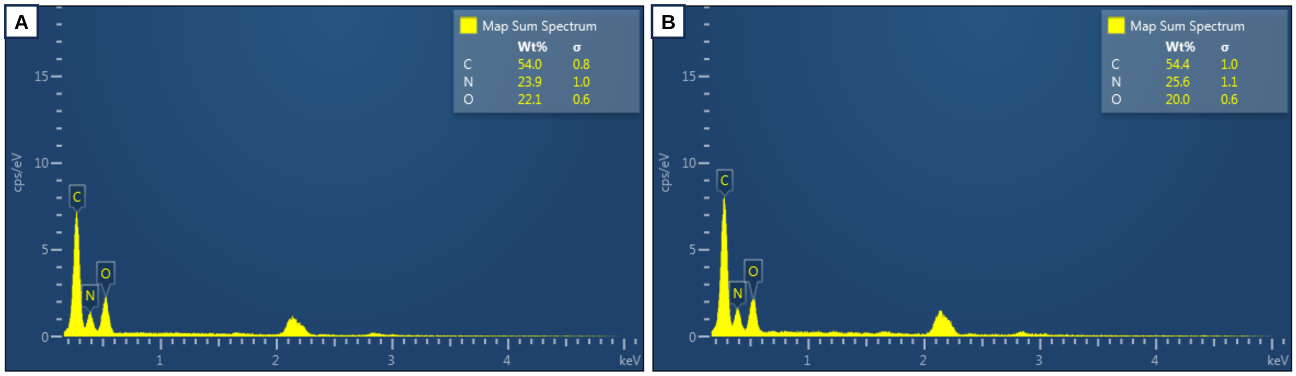

Figure S2. Map sum spectra of (A) BT_1 and (B) BT_5 organohydrogels analyzed with EDS.

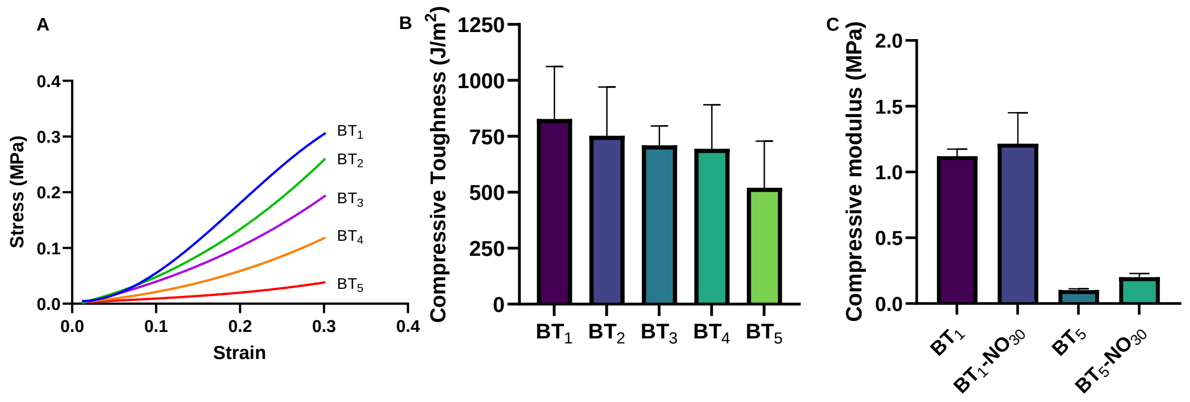

Figure S3. (A) Representative stress-strain curve for gels formulation fitted using non-linear third order polynomial regression. (B) Compressive toughness of gel samples BT_1-BT_5. (C) Compressive modulus of BT_1 and BT_5 gel when compared to BT_1-NO_{30} and BT_5-NO_{30}.

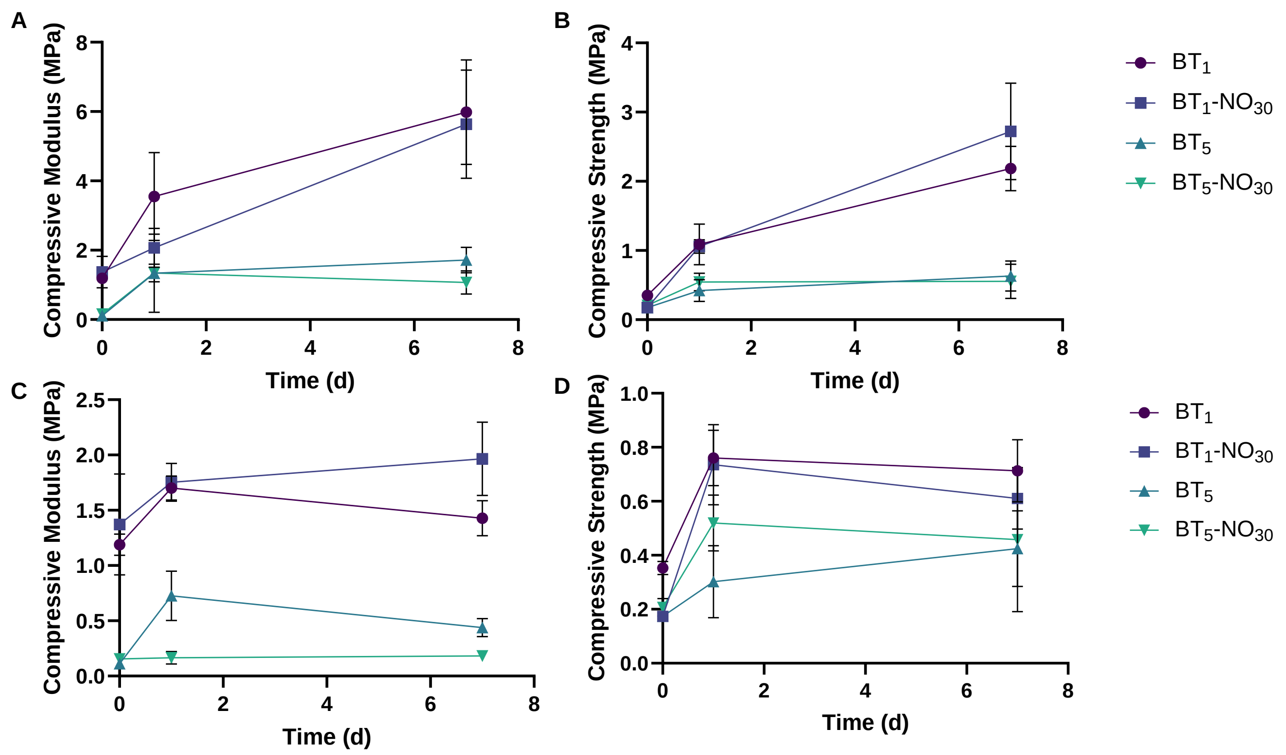

Figure S4. (A) Compressive modulus and (B) compressive strength of samples stored at 37°C for 7d. (C) Compressive modulus and (D) compressive strength of samples stored at 4°C for 7d.

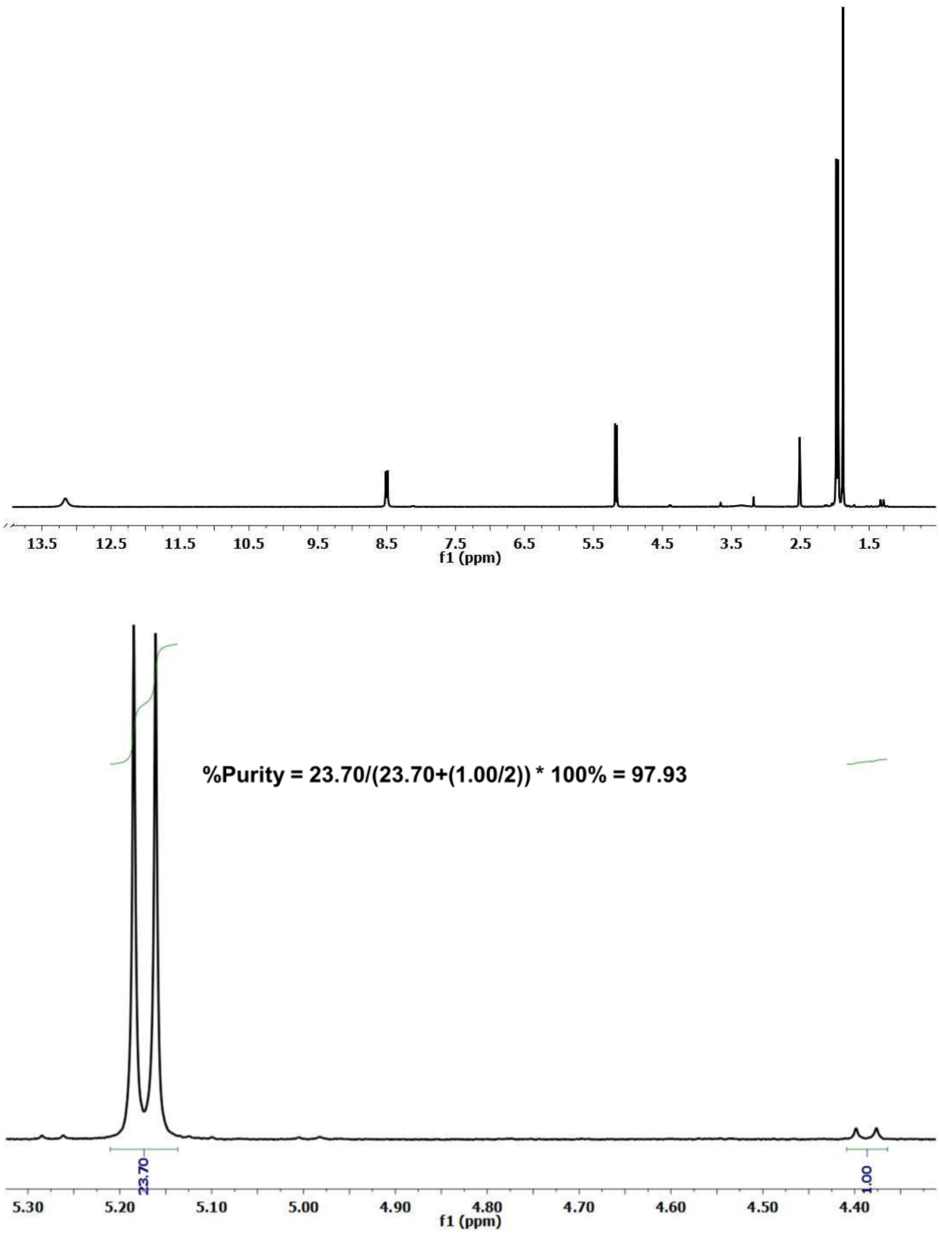

Figure S5. ^{1}H NMR spectra of *S*-nitroso-*N*-acetylpenicillamine (SNAP) confirming the structure and purity.

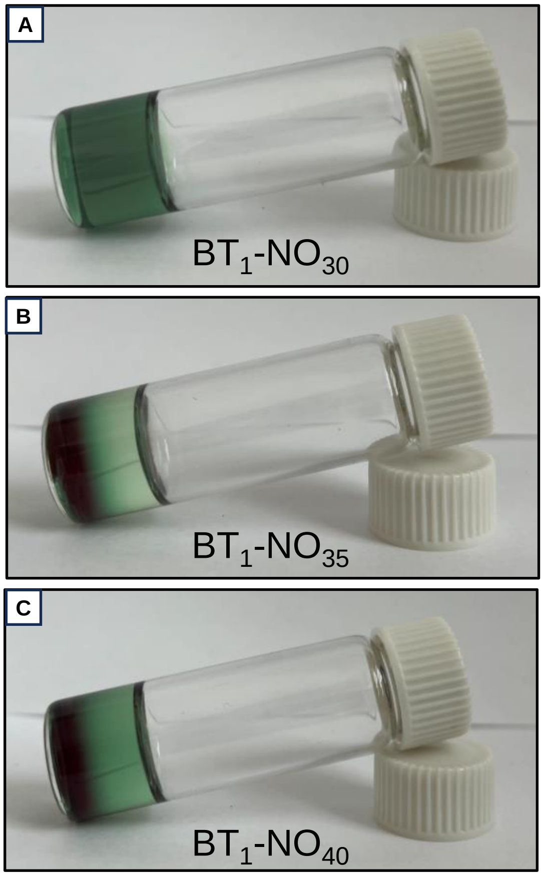

Figure S6. BT_1 organohydrogel with (A) 30 mg/mL, (B) 35 mg/mL, and (C) 40 mg/mL of SNAP blended into the precursor organohydrogel solution.

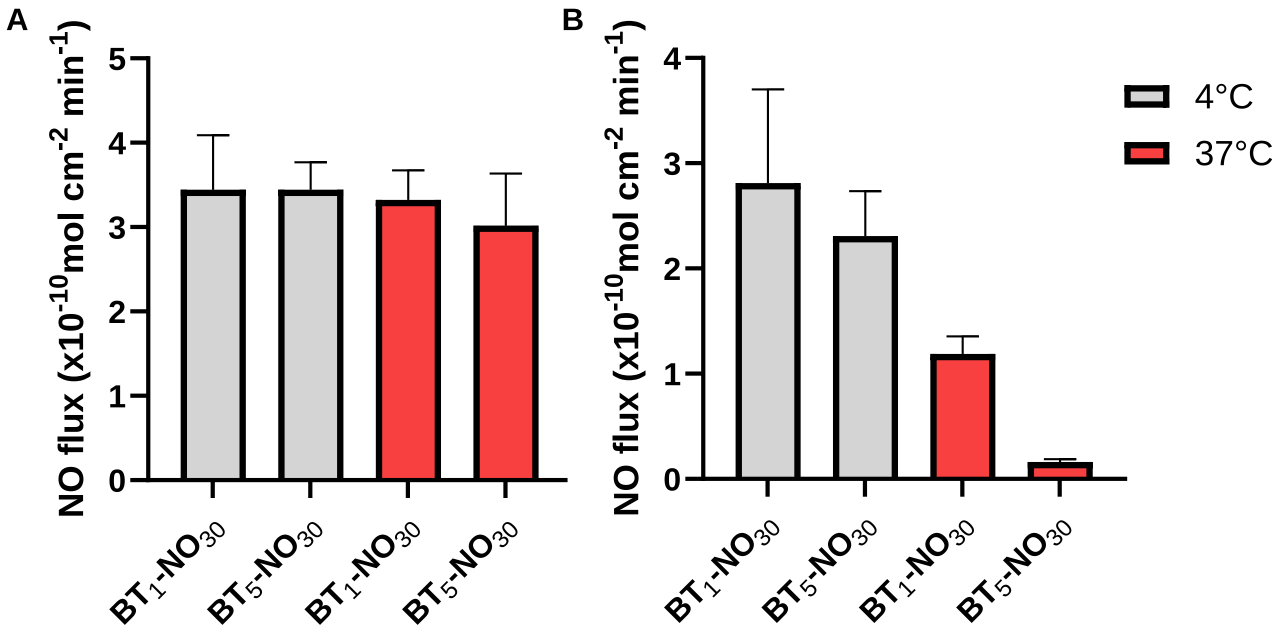

Figure S7. NO release from BT_1-NO_{30} and BT_5-NO_{30} gels after storage at 4°C and 37°C for (A) 24h and (B) 7d.

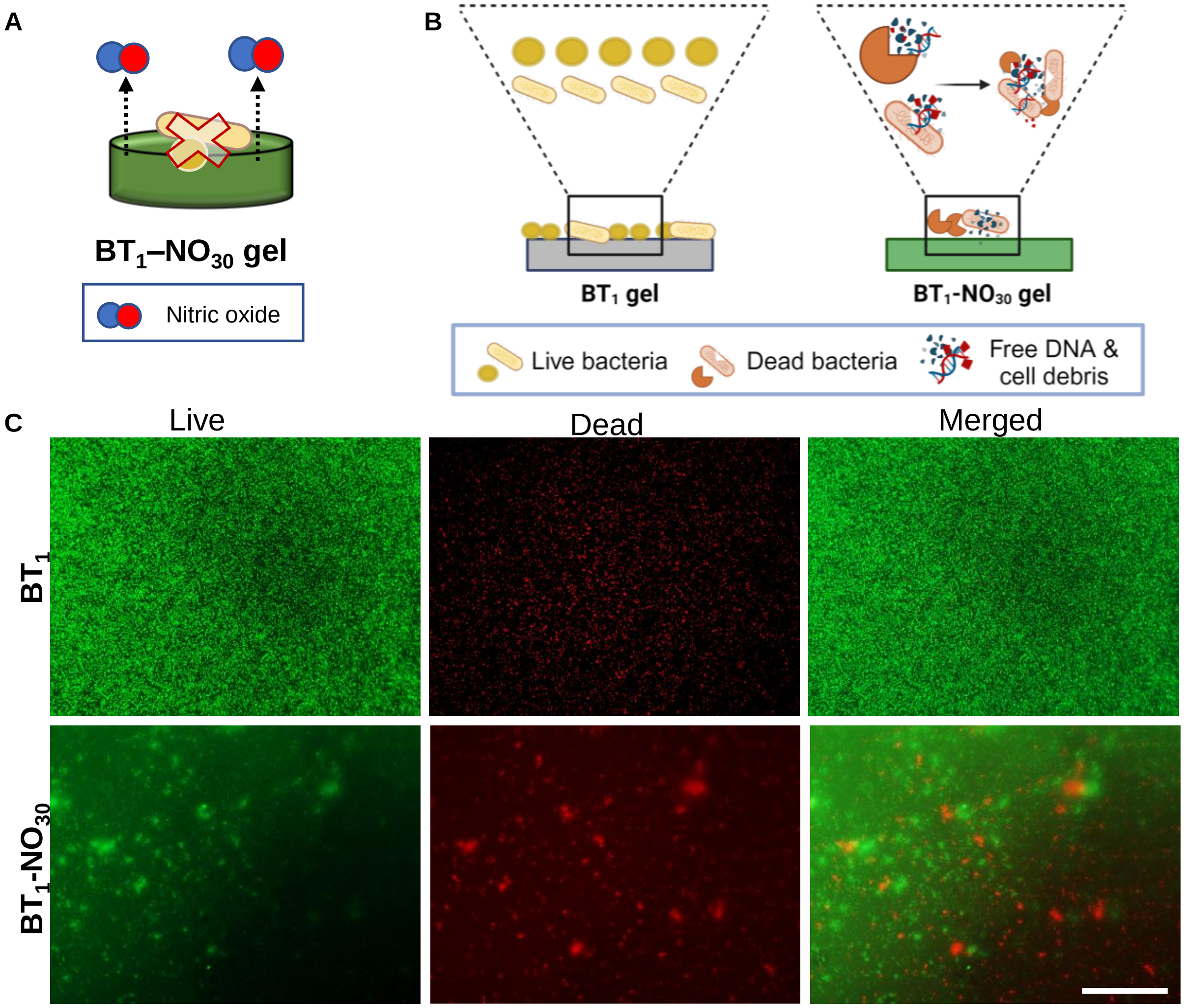

Figure S8. (A) Schematic of NO releasing antibacterial gel sample. (B) Schematic of antibacterial mechanism of NO releasing BT gel. (C) LIVE/DEAD assay showing enhanced antibacterial activity of BT_1-NO_{30} gel against *S. aureus* when compared to BT_1 alone. Scale bar represents 50 μm.

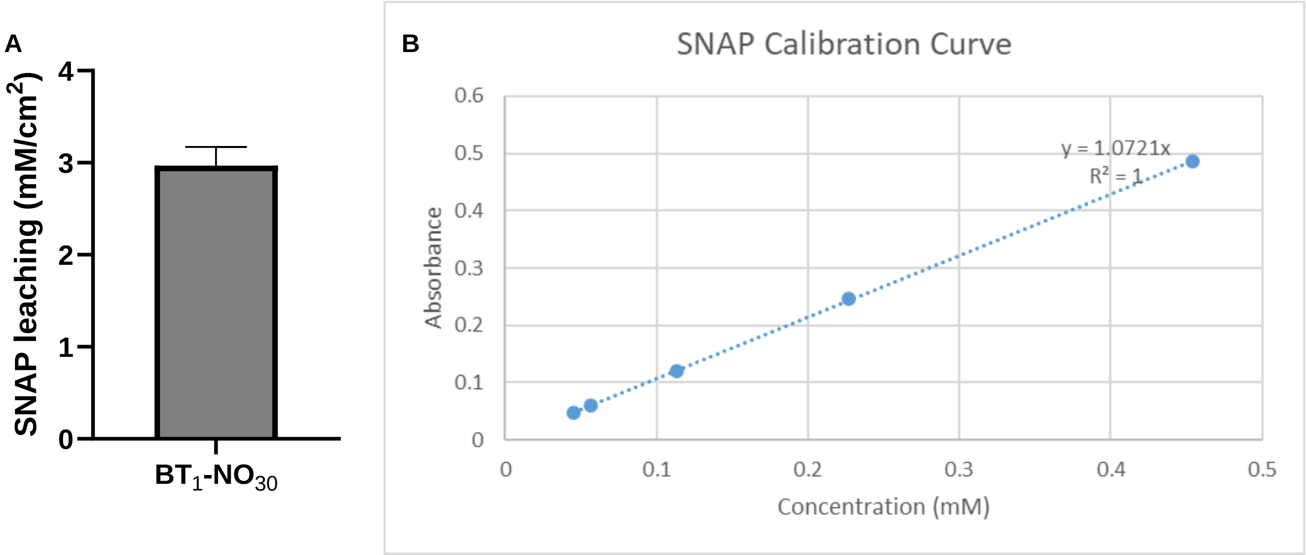

Figure S9. (A) The amount of SNAP leaching from BT_1-NO_{30} gel over 24 h. (B) SNAP calibration curve

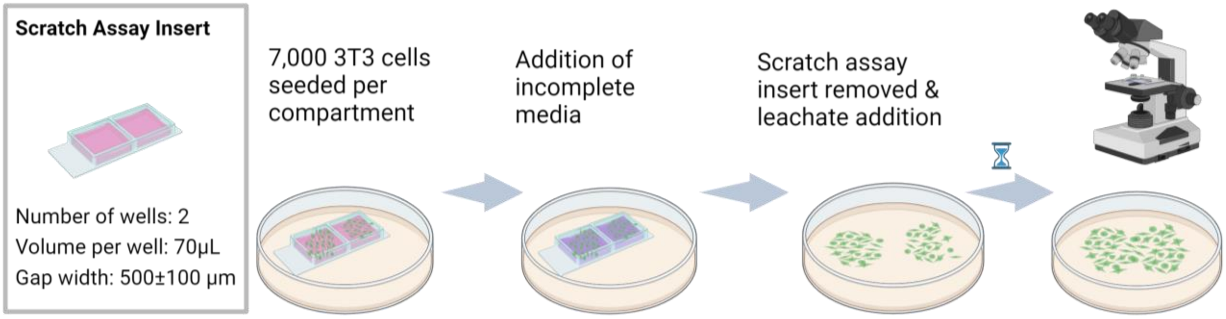

Figure S10. Schematic of the scratch assay experiment performed with 3T3 mouse fibroblast cells.

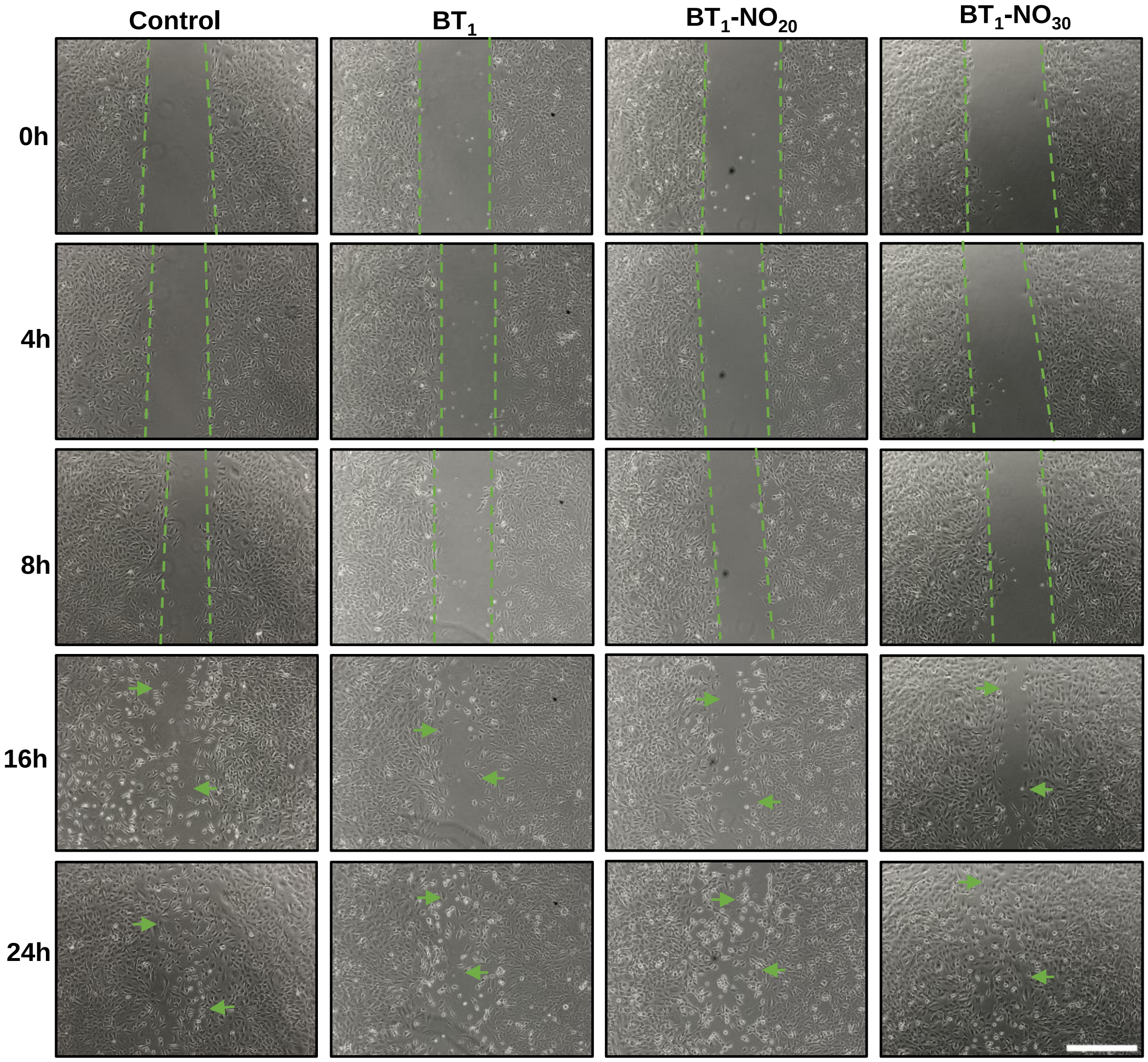

Figure S11. Reduction in scratch area of cells when exposed to different treatment/leachate imaged at various time points. Scale bar represents 500 μm.

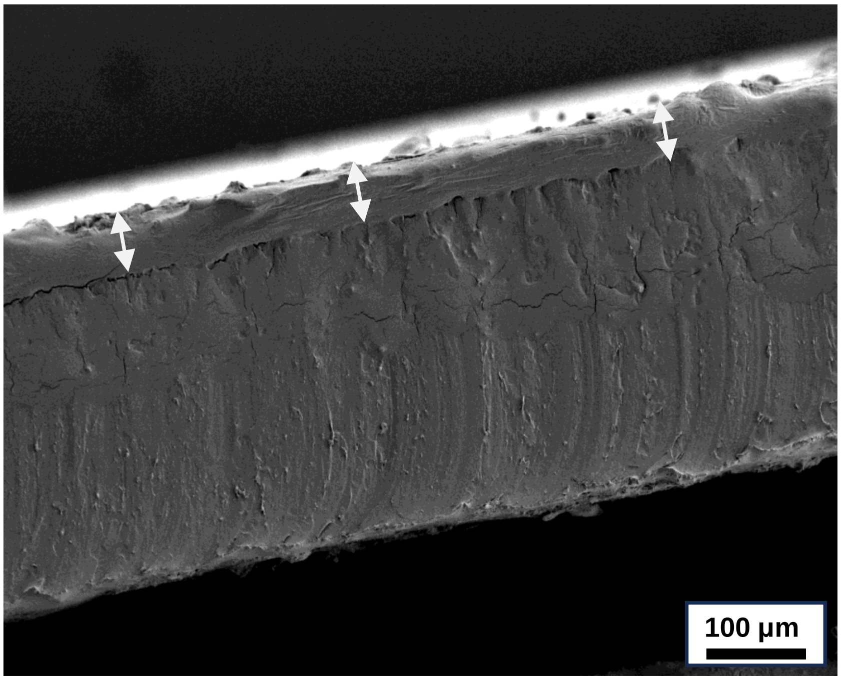

Figure S12. Cross section of BT_1 coated Elasteon substrate observed under SEM. White arrows show the thickness of the coating.

Table S2. Comparison of mechanical properties and biological application of proposed work to current trend in hydrogels and organogels

	Chemistry	Application	Compressive Modulus (MPa)	Cytocompatibility	Antibacterial Analysis	Ref
Organogel	Polyacryl amide (PAAm) + DMSO	Color changing sensors	0.0072	Not performed	Not performed	1
	Lignin + PVA in DMSO	Antifreezing and dehydration gels	0.2-0.5	Not performed	Not performed	2
	Zein + (N-methyl pyrrolidone (NMP) + water) + (DMSO + glycerol formal + water)	Injectable ciprofloxacin-loaded gels for basic 3D printing	0.01	Compatible	ZOI against *S. aureus*	3
	N-lauroyl L-alanine methyl ester + oleic acid + Fe_3O_4 nanoparticles + hydrophobic drug	Thermo responsive organogel for hydrophobic drug delivery	Not performed	Not performed	Not performed	4
	PVA + poly(3,4-ethylenedioxythiophene) + polystyrene sulfonate (PSS) + ethylene glycol (EG) + water	Antifreezing conductive organo-hydrogel	~1	Not performed	Not performed	5
	poly(3,4-ethylene-dioxythiophene) + PSS + PAAm + water	Organogel-based stretchable electronic conductors	0.08	Not performed	Not performed	6
Hydrogel	PVA + PAAm + water + polyhexamethylene guanidine (PHMG) [antibacterial]	Adhesive hydrogel for body sensor and antibacterial activity	Not performed	Compatible	ZOI against *E. coli* and *S. aureus*	7
	Quaternized Chitosan (QCS) + PDA+ PAM	Adhesive, self-healing and antibacterial hydrogel	Not performed	Compatible	Antibacterial against *E. coli* and *S. aureus*	8
Organohydrogel	PVA+ silk nanofibers+ graphitic carbon nitride	Anti-freezing, antibacterial, and conductive strain sensor	0.002 (tensile)	Not performed	Antibacterial against *E. coli* and *S. aureus*	9
	PVA+ chestnut tannin+ nano-silver particles (AgNPs)+ aluminum trichloride+ glycerol+ water	Antibacterial, anti-freezing, and conductive strain sensor	0.03 (tensile)	Compatible	ZOI against *E. coli* and *S. aureus*	10

	Durian cellulose+ glycerol+ water+ epichlorohydrin	Anti-freezing and antimicrobial wound dressing	0.0012 – 0.0136 (tensile)	Compatible	ZOI against *E. coli* and *S. aureus*	[11]
	Branched polyethyleneimine (PEI) + trimethylolpropane triglycidyl ether (TMPGE) + ethanol	NO releasing antibacterial organogel with antifouling properties as coating material	Variable from 0.10- 1.12	Compatible (scratch assay)	Antibacterial against *E. coli* and *S. aureus*	This work

References

(1) Chen, J.; Huang, J.; Zhang, H.; Hu, Y. A Photoresponsive Hydrogel with Enhanced Photoefficiency and the Decoupled Process of Light Activation and Shape Changing for Precise Geometric Control. *ACS Applied Materials & Interfaces* **2020**, *12* (34), 38647-38654. DOI: 10.1021/acsami.0c09475.
(2) Feng, Y.; Yu, J.; Sun, D.; Ren, W.; Shao, C.; Sun, R. Solvent-induced in-situ self-assembly lignin nanoparticles to reinforce conductive nanocomposite organogels as anti-freezing and anti-dehydration flexible strain sensors. *Chemical Engineering Journal* **2022**, *433*, 133202. DOI: https://doi.org/10.1016/j.cej.2021.133202.
(3) Raza, A.; Hayat, U.; Zhang, X.; Wang, J.-Y. Self-assembled zein organogels as in situ forming implant drug delivery system and 3D printing ink. *International Journal of Pharmaceutics* **2022**, *627*, 122206. DOI: https://doi.org/10.1016/j.ijpharm.2022.122206.
(4) Dong, J.; Du, X.; Zhang, Y.; Zhuang, T.; Cui, X.; Li, Z. Thermo/glutathione-sensitive release kinetics of heterogeneous magnetic micro-organogel prepared by sono-catalysis. *Colloids and Surfaces B: Biointerfaces* **2021**, *208*, 112109. DOI: https://doi.org/10.1016/j.colsurfb.2021.112109.
(5) Rong, Q.; Lei, W.; Chen, L.; Yin, Y.; Zhou, J.; Liu, M. Anti-freezing, Conductive Self-healing Organohydrogels with Stable Strain-Sensitivity at Subzero Temperatures. *Angewandte Chemie International Edition* **2017**, *56* (45), 14159-14163. DOI: https://doi.org/10.1002/anie.201708614 (acccessed 2024/01/05).
(6) Lee, Y.-Y.; Kang, H.-Y.; Gwon, S. H.; Choi, G. M.; Lim, S.-M.; Sun, J.-Y.; Joo, Y.-C. A Strain-Insensitive Stretchable Electronic Conductor: PEDOT:PSS/Acrylamide Organogels. *Advanced Materials* **2016**, *28* (8), 1636-1643. DOI: https://doi.org/10.1002/adma.201504606 (acccessed 2024/01/05).
(7) Li, Z.; Xu, W.; Wang, X.; Jiang, W.; Ma, X.; Wang, F.; Zhang, C.; Ren, C. Fabrication of PVA/PAAm IPN hydrogel with high adhesion and enhanced mechanical properties for body sensors and antibacterial activity. *European Polymer Journal* **2021**, *146*, 110253. DOI: https://doi.org/10.1016/j.eurpolymj.2020.110253.
(8) Chen, Y.; Wang, Q.; Li, D.; Mensah, A.; Qiu, Y.; Ke, H.; Wei, Q. Mussel-inspired double cross-linked hydrogels with desirable mechanical properties, strong tissue-adhesiveness, self-healing properties and antibacterial properties. *Materials Science and Engineering: C* **2021**, *120*, 111690. DOI: https://doi.org/10.1016/j.msec.2020.111690.
(9) Bao, S.; Gao, J.; Xu, T.; Li, N.; Chen, W.; Lu, W. Anti-freezing and antibacterial conductive organohydrogel co-reinforced by 1D silk nanofibers and 2D graphitic carbon nitride nanosheets as flexible sensor. *Chemical Engineering Journal* **2021**, *411*, 128470. DOI: https://doi.org/10.1016/j.cej.2021.128470.
(10) Song, B.; Fan, X.; Gu, H. Chestnut-Tannin-Crosslinked, Antibacterial, Antifreezing, Conductive Organohydrogel as a Strain Sensor for Motion Monitoring, Flexible Keyboards, and Velocity Monitoring. *ACS Applied Materials & Interfaces* **2023**, *15* (1), 2147-2162. DOI: 10.1021/acsami.2c18441.
(11) Cui, X.; Lee, J.; Ng, K. R.; Chen, W. N. Food Waste Durian Rind-Derived Cellulose Organohydrogels: Toward Anti-Freezing and Antimicrobial Wound Dressing. *ACS Sustainable Chemistry & Engineering* **2021**, *9* (3), 1304-1312. DOI: 10.1021/acssuschemeng.0c07705.
